# A Guidance for Concomitant Drug Reconciliation Prior to Allogeneic Hematopoietic Cell Transplantation in Children and Young Adults

**DOI:** 10.3389/fped.2021.713091

**Published:** 2021-07-19

**Authors:** Beth Apsel Winger, Susie E. Long, Jordan Brooks, Ashish O. Gupta, Christopher C. Dvorak, Janel Renee Long-Boyle

**Affiliations:** ^1^Division of Allergy, Immunology, and Bone Marrow Transplantation, Department of Pediatrics, University of California, San Francisco, San Francisco, CA, United States; ^2^Division of Hematology and Oncology, Department of Pediatrics, University of California, San Francisco, San Francisco, CA, United States; ^3^Department of Pharmacy, M Health Fairview, Minneapolis, MN, United States; ^4^Department of Clinical Pharmacy, University of California, San Francisco, San Francisco, CA, United States; ^5^Division of Pediatric Blood and Marrow Transplant and Cell Therapy, Department of Pediatrics, University of Minnesota, Minneapolis, MN, United States

**Keywords:** drug–drug interactions, pharmacokinetics, pediatric, hematopoietic cell transplantation, concomitant medication, chemotherapy

## Abstract

Pediatric diseases treated by allogeneic hematopoietic stem cell transplantation (alloHCT) are complex and associated with significant comorbidities and medication requirements that can complicate the transplant process. It is critical to reconcile pre-transplant concomitant medications (pcon-meds) in the weeks prior to alloHCT and to consider the potential for pcon-meds to cause harmful drug-drug interactions (DDIs) or overlapping toxicities with conditioning agents. In this perspective, we describe a systematic process to review pcon-meds and determine the drug modifications needed to avoid DDIs with conditioning regimens. We provide an extensive appendix with timelines for discontinuation or modification of common pcon-meds that patients are taking when presenting to the HCT medical team. The timelines are based on the pharmacokinetic (PK) properties of both the pcon-meds and the planned conditioning medications, as well as anticipated DDIs. They also account for the ages seen at pediatric transplant centers (0–30 years old). Common scenarios, such as when pcon-med discontinuation is not an option, are discussed. Since alloHCT patients are often dependent upon psychiatric medications with problematic DDIs, a table of alternative, non-interacting psychiatric medications is also presented. The appendix provides details regarding how to adjust pcon-meds prior to the start of chemotherapy for children and young adults undergoing alloHCT, however patient-specific circumstances always need to be taken into account. Careful attentiveness to pcon-meds at the time the decision is made to pursue transplant will result in more consistent HCT outcomes, with lower toxicity and increased efficacy of conditioning agents.

## Introduction

Indications for allogeneic hematopoietic cell transplantation (alloHCT) in children and young adults range from malignant disorders such as high-risk leukemia to nonmalignant conditions such as primary immunodeficiencies, hemoglobinopathies, and inherited metabolic disorders (1). Pediatric diseases treated by alloHCT are complex and are often associated with significant comorbidities and medication requirements that can further complicate the transplant process (2, 3). Therefore, it is critical to reconcile the medications that patients are taking when they first present to the HCT medical team, referred to as pre-transplant concomitant medications (pcon-meds), in the weeks leading up to alloHCT. The medication reconciliation process evaluates the potential for pcon-meds to cause harmful drug-drug interactions (DDIs) or exacerbate toxicities with conditioning agents. DDIs can either increase or decrease the exposure of various conditioning agents, and thereby negatively impact outcomes by contributing to severe drug-related toxicities, graft rejection, and disease relapse.

Pcon-meds vary widely in their potential to cause harmful DDIs with alloHCT conditioning. The detailed mechanisms by which DDIs result in altered pharmacokinetics (PK) and pharmacodynamics (PD) are well described elsewhere in the literature (4). The goals for this perspective are: (a) to provide an overview of ideal medication changes prior to the start of conditioning, and, (b) to provide a framework for assessing the potential impact of pcon-meds on HCT conditioning agents. As an extensive resource, Appendix 1 lists common pcon-meds, provides recommendations for the optimal timing for discontinuation of pcon-meds in relation to the start of conditioning chemotherapy, and also provides potential medication alternatives for commonly used psychiatric medications that have problematic DDIs.

## Background

### Impact of Pcon-Meds on Cytochrome P450 (CYP450) Enzymes

**Figure 1:**
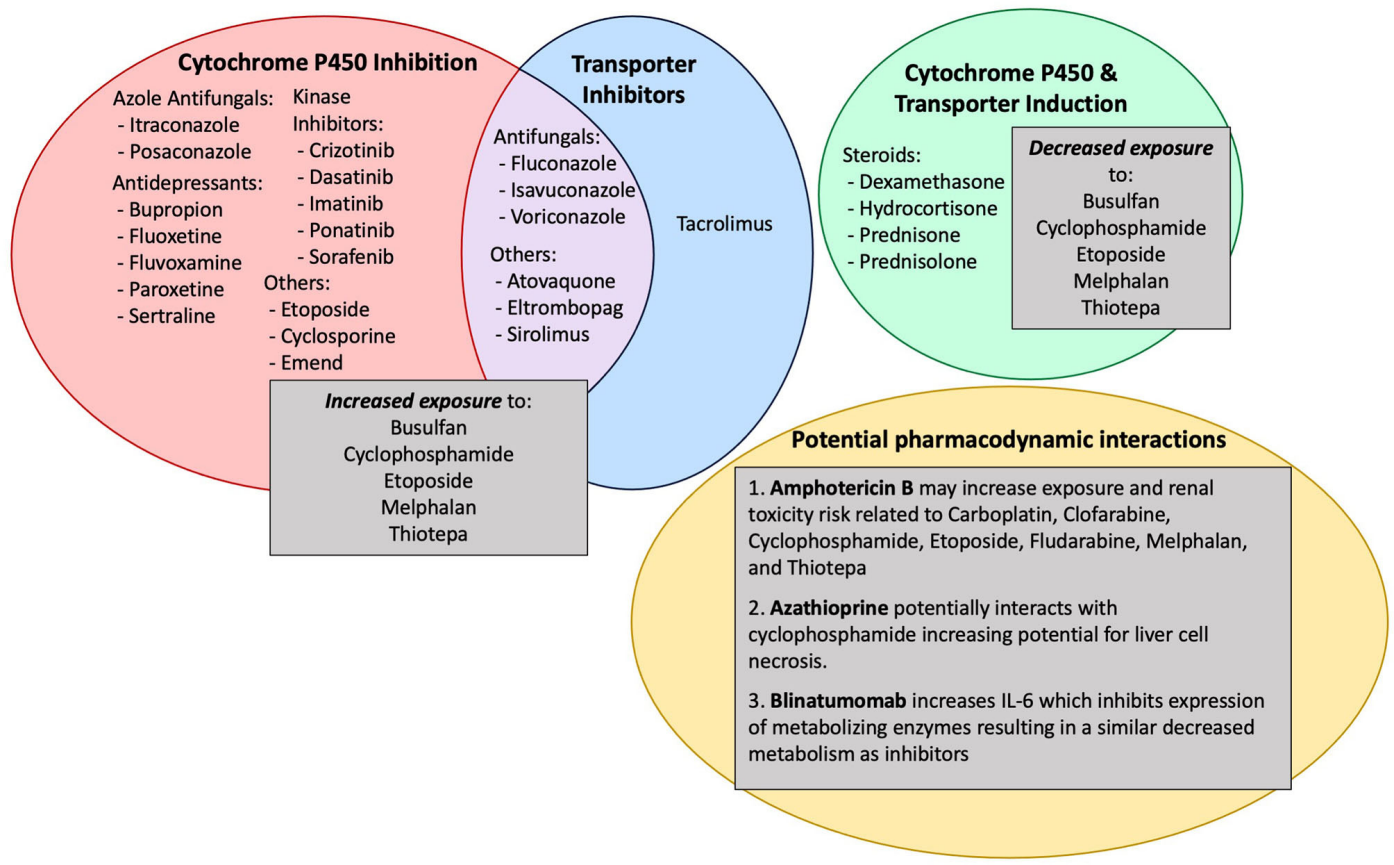
Common pcon-meds, mechanisms by which they cause drug-drug interactions and the impact on conditioning agents.

Most clinical trials evaluating new therapies enroll a homogeneous population with strict eligibility criteria for limiting DDIs. Thus, the potential effects of pcon-meds are rarely evaluated formally in a real-world setting. Instead, the potential for a pcon-med to alter the PK or PD of a conditioning agent is often extrapolated based on the known impact of similar drugs “in-class” or based on individual case reports. For example, the potential for pcon-meds to alter drug metabolism outside the setting of alloHCT is often well-understood and thus applied to how these medications may impact conditioning agents used during alloHCT. Pcon-meds most commonly alter the metabolism of conditioning agents via induction or inhibition of cytochrome p450s or via interference with drug transporters (Figure 1) (4). This can have a profound effect on the exposure and efficacy of conditioning agents. If metabolism is induced, the conditioning agent can be less effective; if metabolism is inhibited, the conditioning agent can have increased toxicity. Aside from busulfan, therapeutic drug monitoring is not routine for conditioning agents, therefore drug exposure cannot be tracked in real-time and the dose of the conditioning agent cannot be adjusted to counteract the impact of interfering pcon-meds. Therefore, it is critical to be aware of the potential impact of pcon-meds on metabolism with enough time to make medication adjustments prior to alloHCT conditioning and avoid DDIs whenever possible. When the clinical scenario makes it impossible to avoid a DDI, awareness of the potential for a DDI can direct appropriate clinical monitoring for toxicity.

The enzymatic system responsible for the majority of phase one metabolism leading to elimination of both endogenous substrates and xenobiotics is the hepatic cytochrome P450 (CYP450) system (5). There are multiple mechanisms by which CYP450 enzymes can be modulated by medications (4). The mechanisms include direct competitive inhibition at the CYP450 active site, induction of CYP450 protein synthesis, and disruption of CYP450 transcription, translation and/or post-translational processing (6). The mechanism of CYP450 dysregulation dictates the time it will take for the enzyme to recover, and is complicated by the fact that different CYP450 isoforms have different half-lives (6). In addition, CYP450 synthesis is a zero order process (meaning not dependent on the concentration of CYP450 enzyme in the body), while CYP450 degradation is a first order process (meaning it is dependent on the concentration of enzyme in the body) (6–8). This is an important difference because it means that synthesis and degradation of CYP450 enzymes often do not occur directly in parallel, and that if the system is disrupted, time is required to re-establish a steady-state level of enzymatic expression.

For pcon-meds that are competitive inhibitors of CYP450 function, the elimination half-life of the drug determines the amount of time that the CYP is inhibited, leading to variable, drug-specific durations in DDI effect (9). Based on basic PK principles, it is well-known that discontinuing competitive CYP inhibitors 5–7 elimination half-lives prior to the start of conditioning will ensure the majority of medication is cleared from systemic circulation. In contrast, CYP450 recovery after discontinuation of enzyme inducers requires the liver to re-establish normal cellular enzyme levels (9). This process takes longer because additional enzyme that has accumulated needs to be degraded. The limited data available suggest that waiting 14 days after stopping a CYP450 inducer should result in ~90% enzyme recovery (9). This concept is not specifically addressed in the appendix, but should be considered by clinicians.

## Materials and Methods

This section describes a systematic process of reviewing pcon-meds and determining what drug modifications are recommended to avoid DDIs with conditioning agents. The process is initiated at the time the decision is made to pursue alloHCT in the weeks to months leading up to pre-transplant conditioning. All medications are reviewed and confirmed for the dose, dose frequency, indication, and potential for DDIs with the anticipated alloHCT conditioning agents. Pcon-meds with well-established or highly suspected DDIs are discontinued whenever possible based on the patients' disease state and medical needs. Appendix Table 1A, a tool developed by the authors to streamline the medication review process, has specific guidelines for discontinuation of commonly used pcon-meds based on DDIs with conditioning agents used in alloHCT (specifically: busulfan, carboplatin, clofarabine, cyclophosphamide, etoposide, fludarabine, melphalan, and thiotepa). The pcon-meds in the table were selected based on input from pharmacists and physicians at UCSF Benioff Children's Hospital and the University of Minnesota Masonic Children's Hospital. In the table, the “Standard Stop Time” was determined by the 5–7 half-lives necessary to clear an offending agent and the impact of the pcon-med on metabolism of the conditioning agents. If not clinically feasible for the patient to stop the medication within the “Standard Stop Time”, a “Minimum Stop Time” is included. In Appendix Table 1A, the “Minimal Stop Time” is the time in days required to limit or minimize the most significant/harmful DDIs and was determined based on the drug clearance of the offending agent and the DDIs present. The timeline for stopping a medication is always rounded up or down in days to maximize patient comprehension and compliance prior to admission. Additionally, the timing for discontinuation of medications does not include the administration of serotherapy prior to the start of cytotoxic chemotherapy given the limited evidence for DDIs with monoclonal antibodies (10).

## Results and Discussion

### When Discontinuation Is Not an Option

Discontinuation of a pcon-med may not be possible for a variety of reasons, including risk of toxicities associated with rapid drug discontinuation, risk of inflammation which may contribute to graft rejection, and risk of relapse of the underlying disease. Certain drugs, such as antidepressants, anticonvulsants and steroids, need to be tapered or converted to alternate agents rather than abruptly stopped to limit symptoms of drug withdrawal (11, 12). For antidepressants and anticonvulsants, coordination with the prescribing provider to develop an appropriate plan for tapering the drug and/or transitioning to another agent is critical for establishing a clear discontinuation timeline, maximizing patient compliance and safety, and ensuring the drug is discontinued by the start of conditioning. For corticosteroids, several forms of steroids (e.g., prednisone and dexamethasone) can lead to enzyme induction which will decrease the activation of cyclophosphamide and thiotepa and can increase the clearance of busulfan (2). Given this effect can be dose-dependent, an attempt to lower the total daily dose of steroids is routinely considered prior to HCT, and conversion of other steroids to hydrocortisone is recommended at least a week prior to start of conditioning, unless methylprednisolone is planned for GVHD prophylaxis. However, for patients with severe, refractory hemophagocytic lymphohistiocytosis (HLH) whose disease is uncontrollable off steroids, a steroid taper prior to HCT may not be possible. For such patients, acceptance of the DDIs described above may be required in order to maintain the patient in a clinical condition acceptable for HCT. In this scenario, implementation of a rapid steroid taper can generally commence after the start of immunoablative conditioning and serotherapy. One final example is a patient with chemo-refractory, CD19-negative acute lymphoblastic leukemia who only achieves remission with inotuzumab, a drug ideally discontinued 3 months prior to transplant due to the risk of veno-occlusive disease. In this scenario, discontinuing inotuzumab closer to transplant (e.g., 2–3 weeks) may be the only way to maintain sufficient disease control to get to definitive therapy. Therefore, some centers would accept the risk of organ toxicity over the risk of leukemia relapse. There are many other examples of situations in which the ideal discontinuation timeline is not an option. In these situations the use of busulfan as the primary alkylator is an attractive option since both model-based dosing and therapeutic drug monitoring of busulfan can overcome drug interactions between busulfan and pcon-meds, enabling optimal exposure.

### Considerations for Drug Formulation

Absorption can vary during the post-transplant period secondary to mucositis or intestinal graft verses host disease. Therefore, transition off oral medications is important to consider pre-transplant. Particularly following myeloablative conditioning, patients are routinely unable to swallow pills, which is a challenge for medications only available in pill form. Extended-release formulations do not allow for splitting or crushing and may not be available in an IV form. For example, as discussed above, many alloHCT patients are dependent upon antidepressants that are only available in pill form. If possible, under the guidance of the prescribing provider, tapering and discontinuation of the patient's current antidepressant should be performed in the weeks prior to alloHCT (Appendix Table 2A).

### Antimicrobials

Several of the “azoles” are well-known for their role in DDIs. Itraconazole, voriconazole and posaconazole are considered significant inhibitors of CYP450 enzymes and have interactions with several of the conditioning agents used for pediatric HCT, as well as interactions with many other pcon-meds (2, 13, 14). Studies have shown that azoles, for example fluconazole verses itraconazole, differ in their impact on metabolic enzymes (15). To minimize any impact, discontinuing all azoles except for fluconazole at least 7 days prior to the start of conditioning is recommended. Echinocandins (e.g., caspofungin) and/or amphotericin B may be used as alternatives for patients who require anti-fungal therapy during conditioning, though amphotericin B must be used carefully in patients with renal impairment. In contrast to anti-fungal medications, anti-viral medications and antibiotics used for pneumocystis (PCP) prophylaxis (trimethoprim-sulfamethoxazole, dapsone) have few DDIs. These agents can be safely continued through conditioning if needed.

### Anti-neoplastic Medications

Targeted therapies, especially tyrosine kinase inhibitors (TKIs), are well-known for having problematic DDIs (16). Based on established PK profiles most require discontinuation over a week prior to the start of conditioning. Other anti-neoplastic agents commonly used for leukemia control prior to transplant (e.g. azacytidine, cytarabine, 6-mercaptopurine (6MP), 6-thioguanine (6TG), vincristine) have minimal DDIs. Although these agents pose little to no risk for altered PK of conditioning agents, stopping a week prior to conditioning may be considered because of concerns for overlapping drug-related toxicity (e.g., hepatic toxicity) that may occur with combination conditioning. Other chemotherapies, such as cytarabine and etoposide do not have the same concerning overlapping toxicities and can be discontinued 24–48 h prior to conditioning.

For intrathecal (IT) therapies, it is recommended to stop IT cytarabine at least 7 days prior to conditioning and IT methotrexate 14 days prior to conditioning. This conservative approach is based on limited formal drug studies evaluating the effects of IT drug administration and DDIs (17) and a theoretical concern that IT chemotherapy could make patients more susceptible to central nervous system (CNS) toxicity by disrupting the blood-brain barrier.

### Interactions Between Drugs and Total Body Irradiation

Drug interactions with TBI are not well defined, however there is some data evaluating changes in the permeability of the blood brain barrier following total body irradiation (TBI) (18, 19). The data available from rodent models demonstrates an increase in the CNS permeability to both endogenous and exogenous substances following both moderate and low doses of TBI. The CNS permeability is most significant at 24–48 h post-TBI (18, 19). Given the sparse data and limited understanding of the potential risk, limiting certain pcon-meds with known neurologic toxicity (e.g., IT chemotherapy), regardless of the conditioning regimen, in the days prior to TBI is recommended.

Additionally, TBI has been linked to increased risk of veno-occlusive disease (VOD), particularly when used in combination with an alkylator, in which case the risk of VOD is dependent on the dose of the alkylator (20, 21). Thus, for all patients receiving TBI, it is important to limit hepatotoxic pcon-meds (e.g., fluoxetine, paroxetine, and isoniazid) and discontinue such medications prior to transplant whenever possible.

### Other Considerations

In addition to analyzing DDIs between pcon-meds and conditioning agents, clinicians should also review patient related risk factors when recommending alternative medications. These include special attention to patient allergies, and patient use of illicit substances, herbal medications, essential oils and/or nutritional supplements. Special attention should be made for very young pediatric patients (<1 year), patients that weigh <10 kg and/or obese patients. Adjustments may also be necessary for patients with renal dysfunction, hepatic dysfunction or cardiac dysfunction (e.g., prolonged QTc). When recommending supportive medications, clinicians should also consider patient-specific preferences. These recommendations are outside of the scope of the paper, however still important to address with both the patient and the HCT medical team prior to starting conditioning.

## Conclusion

The tables included in the Appendix of this manuscript provide detailed guidance regarding how to adjust and discontinue pcon-meds for pediatric patients prior to conditioning for alloHCT. However, as outlined above, the considerations that we have incorporated into this table are extensive and detailed, and the table is a guideline that requires provider interpretation and clinical expertise individualized to each patient. As outlined in this perspective, careful attentiveness to pcon-meds at the time the decision is made to pursue transplant will result in more consistent HCT outcomes, with lower toxicity and/or increased efficacy of conditioning agents.

## Data Availability Statement

The original contributions presented in the study are included in the article/Supplementary Material, further inquiries can be directed to the corresponding author.

## Author Contributions

BAW, SL, JB, AG, CD, and JLB conceived of the manuscript, selected the drugs to include in the main table (Supplementary Table 1A), manuscript and generated the table. All authors contributed to the article and approved the submitted version.

## Conflict of Interest

The authors declare that the research was conducted in the absence of any commercial or financial relationships that could be construed as a potential conflict of interest.
