# Supplementary material for: A Guidance for Concomitant Drug Reconciliation Prior to Allogeneic Hematopoietic Cell Transplantation in Children and Young Adults
Source: Front Pediatr. 2021 Jul 19;9:713091. doi: 10.3389/fped.2021.713091 (PMC8326409; doi:10.3389/fped.2021.713091)
Supplement: Supplementary file 1 [file Data_Sheet_1.docx]

Supplementary Material

**A Guidance For Concomitant Drug Reconciliation** **Prior To Allogeneic Hematopoietic Cell Transplantation In Children And Young Adults**

Beth Apsel Winger, MD, PhD, Susie E. Long, PharmD, Jordan Brooks, PharmD, Ashish O. Gupta MD, MPH, Christopher C. Dvorak, MD, Janel Long-Boyle, PharmD, PhD

Correspondence to: [beth.winger@ucsf.edu](mailto:beth.winger@ucsf.edu)

This supplementary material includes:

Appendix 1: Table 1A, Table 1B

**Appendix 1.**

**Table 1A.** **Pre-transplant Concomitant Medications (Pcon-meds) and their Drug-Drug Interactions with Conditioning Regimens**

Timing for discontinuation of medications *does not include* the administration of serotherapy prior to conditioning. Cytotoxic chemotherapy considered for DDIs: busulfan, carboplatin, clofarabine, cyclophosphamide, etoposide, fludarabine, melphalan, and thiotepa. Drug Class: 1 – Antimicrobial, 2 – Immunosuppressive, 3 – Anticancer, 4 – Antidepressants, or 5 – Supportive Care. Standard Stop Time is the time in days prior to the start of conditioning needed to clear the pcon-med from systemic circulation (e.g. 5-7 half-lives). The Minimum Stop Time is the time in days prior to the start of conditioning required to limit the most significant/harmful DDIs considering drug clearance of offending agent.

Abbreviations: PO – per oral, IV – intravenous; IT – Intrathecal; IM – Intramuscular; SQ – subcutaneous; INH – inhaled; CYP – cytochrome P450; GST – glutathione, P-gp - P-glycoprotein; UGT – Uridine 5'-diphospho-glucuronosyltransferase; PK – pharmacokinetic; PD – pharmacodynamic; hr – hours; GI – gastrointestinal; CrCl – creatinine clearance; AUC – area under the curve.

| **The standard and minimum stop times provided are considered optimal for limiting drug-drug interactions.**  **Each patient must be evaluated individually for what is feasible given his/her disease status and clinical needs.** | | | | | | | |
| --- | --- | --- | --- | --- | --- | --- | --- |
| **Drug Class** | **Pcon-Med**  **(route)**  **(reference)** | **Metabolic Pathway** | **PK/PD interactions (general & with conditioning agents)** | | **Half-life** | **Standard Stop Time** | **Minimum Stop Time** |
| 3 | 6-Mercaptopurine  (PO)  (1-3) | - Hepatically metabolized via oxidation and methylation by thiopurine methyltransferase to inactive metabolites  - Excreted in the urine as unchanged drug and metabolites (~46%)  - Not a CYP inducer or inhibitor | PK: None  PD:  Hepatotoxicity Myelosuppression | | Children: 1.2 hr  (0.4-3.3 hr)  Adult: 1.3 hr | 7 days | 3 days |
| 3 | 6-Thioguanine  (PO)  (3, 4) | - Hepatically metabolized via thiopurine methyltransferase to active metabolites and inactive metabolites  - Excreted in the urine as active metabolites  - Not a CYP inducer or inhibitor | PK: None  PD:  Hepatotoxicity Myelosuppression | Children: 2 hr  (0.8-6.2 hr)  Adult: 1.33 hr | | 7 days | 3 days |
| 5 | Acetaminophen  (IV, PO)  (5-9) | - Hepatically metabolized via glucuronidation, sulfation and by CYP2E1 to NAPQI, which is subsequently conjugated rapidly with glutathione to inactive metabolites  - Excreted in the urine as unchanged drug (<5%) and inactive metabolites (80%)  - Not a CYP inducer or an inhibitor | PK: Busulfan  PD: None | Neonate: 7 hr  (4-10 hr)  Infant: ~4 hr  (1-7 hr)  Children: 3 hr  (2-5 hr)  Adolescent: ~3 hr  (2-4 hr)  Adult: ~2 hr  (2-3 hr)  may be slightly prolonged in severe renal insufficiency (CrCl <30 mL/minute): 2-5.3 hr | | 1 day if busulfan is used for conditioning and acetamino-phen is not required as a pre-medication for ATG | May continue through conditioning |
| 1 | Acyclovir  (IV, PO)  (10, 11) | - Prodrug converted by viral enzymes to acyclovir monophosphate, and further converted to diphosphate then triphosphate (active form) by cellular enzymes  - Excreted in the urine as unchanged drug and active metabolites  - Inhibitor of CYP1A2 (weak) | PK: None  PD: Renal toxicity (IV) | Children:  2.36-3.8 hr  Adult: 2.5-3.5 hr | | May continue through conditioning | |
| 5 | Allopurinol  (IV, PO)  (12, 13) | - Hepatically metabolized via oxidation to active metabolites  - Excreted in the urine (80%) as unchanged drug and active metabolite and in the feces (20%)  - Not a CYP inducer or inhibitor | PK: None  PD: Renal toxicity | Children:  Neonate ~2.5 hr (parent compound)  Adult: 1.5 hr (parent compound) 15 hr (active metabolite) | | May continue through conditioning | |
| 5 | Amlodipine  (PO)  (14) | - Hepatically metabolized to inactive metabolites  - Excreted in the urine as unchanged drug and inactive metabolites  - Inhibitor of CYP3A4 (weak)  - Substrate of CYP3A4 (strong) | PK: None  PD: None | Children: none  Adult: 30-50 hr | | May continue through conditioning | |
| 1 | Amphotericin B  (IV)  (15, 16) | - Metabolic pathway unknown; assumed to be renal | PK:  Carboplatin  Clofarabine  Cyclophosphamide  Etoposide  Fludarabine  Melphalan  Thiotepa  PD: Renal toxicity | Children: 45 hr  Adult: 15 days | | May continue through conditioning | |
| 5 | Aprepitant  (IV, PO)  (17, 18) | - Extensive hepatic metabolism via CYP3A4, CYP1A2 and CYP2C19 to weakly active metabolites  - Excreted via metabolism  - Inhibitor of CYP3A4 (moderate)  - Inducer of CYP2C9 (weak) with prolonged use | PK:  Busulfan  Cyclophosphamide  Etoposide  Melphalan  Thiotepa  PD: None | Children: 2-5 hr  Adult: 9-13 hr | | If consistent use for > 1 week, 14 days | If minimal/ inconsistent use, 2 days |
| 1 | Atovaquone  (PO)  (19, 20) | - Not metabolized/metabolic pathway unknown  - Excreted in the bile as unchanged drug  - Possible enterohepatic recirculation  - P-gp/ABCB1 inhibitor | PK: Etoposide  PD: None | Children: Unknown  Adult:  33.6–100.2 hr | | 2 days | May continue through conditioning |
| 3 | Azacitidine  (IV, PO)  (21) | - Prodrug is converted to active triphosphate and deoxy- triphosphate forms by cellular enzymes  - Excreted in the urine  - Not a CYP inducer or inhibitor | PK: None  PD:  Myelosuppression | Children: Unknown  Adult IV/SQ: 4 hr  Oral: 0.5 hr | | May continue through conditioning | |
| 2 | Azathioprine  (PO)  (22) | - Prodrug is converted to 6-mercaptopurine via glutathione reduction  - Further hepatically metabolized to active and inactive metabolites  - Excreted in the urine primarily as metabolites  - Not a CYP inducer or inhibitor | PK: Cyclophosphamide  PD:  Hematologic toxicities Hepatotoxicity  GI toxicity | Children: 2 hr  (0.8-6.2 hr)  Adult: 1.33 hr | | 1 day | 1 day |
| 1 | Azithromycin  (IV, PO)  (23) | - Hepatically metabolized to inactive metabolites  - Excreted in the bile as unchanged parent drug  - P-gp/ABCB1 inhibitor  - Substrate of CYP3A4 (weak) | PK: Etoposide  PD: None | Children: 54.5 hr  Adult: 68-72 hr | | May continue through conditioning | |
| 3 | Blinatumomab  (IV)  (24-28) | - Metabolic pathway unknown | PK:  Busulfan  Cyclophosphamide  Etoposide  Melphalan  Thiotepa  PD: None | Children: 2 hr  (0.6-3.8 hr)  Adult: 2.1 hr  (0.7-3.5 hr) | | 14 days | 14 days |
| 3 | Bortezomib  (SQ, IV)  (29-31) | - Hepatically metabolized primarily via CYP2C19 and CYP3A4 and to a lesser extent CYP1A2 to inactive metabolites; forms inactive metabolites via deboronization followed by hydroxylation  - Eliminated in the urine and the bile  - Substrate of CYP3A4 (strong), CYP1A2 (weak), CYP2C19 (weak), CYP2C9 (weak), and CYP2D6 (weak) | PK: None  PD: Neutropenia, thrombocytopenia | Children:  Beta 1.58 hr  Terminal 100 hr  Adult:  Single dose: 9-15 hr  Multiple doses (1 mg/m^2)^: 40-193 hr  Multiple doses (1.3 mg/m^2^): 76-108 hr | | May continue through conditioning | |
| 4 | Bupropion  (PO)  (32-34)  *(see alternative options in*  *Table 2)* | - Extensive hepatic metabolism via CYP2B6 and non-CYP mediated metabolism to active metabolites which range from 20-50% activity. Also undergoes oxidation and glycine conjugation to form its major urinary metabolite  - Excreted in the urine (87%) and the feces (10%)  - Inhibitor of CYP2D6 (strong), OCT2 | PK:  Busulfan  Cyclophosphamide  Etoposide  Melphalan  Thiotepa  PD: Seizure | Adolescent:  12.1 hr  Adult: 19-21.3 hr | | Consult with prescribing provider to develop adequate taper resulting in discontinuation at least 7 days prior to conditioning | |
| 5 | Carbamazepine  (PO)  (35, 36) | - Hepatically metabolized via CYP3A4 to epoxide active metabolite, followed by epoxide hydrolase to transdiol inactive metabolite  - Excreted in the urine (72%) and feces (28%) as inactive metabolites  - Inducer of CYP3A4 (strong), CYP2B6 (strong), CYP2C9 (weak)  - Induces P-gp/ABCB1 | PK:  Busulfan  Cyclophosphamide  Etoposide  Melphalan  Thiotepa  PD: None | Children & Adolescent:  3.1-20.8 hr  Adult: 12–17 hr | | Consult with prescribing provider to develop adequate taper resulting in discontinuation at least 14 days prior to conditioning | |
| 1 | Caspofungin  (IV)  (37) | - Hepatically metabolized via hydrolysis and N-acetylation, spontaneous degradation to inactive metabolites  - Excreted in the urine and feces as inactive metabolites  - Not a CYP inducer or inhibitor | PK: None  PD: None | Children:  3-23 month: 8.8 hr  2-11 years: 8.2 hr  12-17 years: 11.2 hr  Adult: 11-23.5 hr | | May continue through conditioning | |
| 1 | Cefepime  (IV)  (38) | - Minimally hepatically metabolized to inactive metabolites  - Excreted in the urine as unchanged drug (85%)  - Not a CYP inducer or inhibitor | PK: None  PD: Renal toxicity  Neurotoxicity | Children: unknown  Adult: 2-2.3 hr | | May continue through conditioning | |
| 5 | Celecoxib  (PO)  (39) | - Hepatically metabolized via CYP2C9 to inactive metabolites  - Excreted in the feces and urine as inactive metabolites  - Inhibitor of CYP2D6 (weak)  - Substrate of CYP2C9 (strong), CYP3A4 (weak) | PK: None  PD: Renal toxicity  Cardiovascular &  thrombotic events  Anemia | Children: 11 hr  Adult: ~11 hr | | 3 days | May continue through conditioning |
| 5 | Cholecalciferol  (PO)  (40) | - Prodrug converted to active forms (25(OH)D and 1,25(OH)2D via sequential hepatic and renal hydroxylation  - Excreted in the bile and feces  - Not a CYP inducer or inhibitor | PK: None  PD: None | Children: unknown  Adult:  Calcidiol: 2-3 weeks  Calcitriol: ~4 hr | | May continue through conditioning | |
| 5 | Crizanlizumab  (IV)  (41) | - Metabolism not fully established. Expected breakdown into small peptides and amino acids via protein catabolic pathways  - Excreted in the bile  - Not a CYP inducer or inhibitor | PK: None  PD: None | Children: Unknown  Adults: 7.6 days (sickle cell disease), 10.6 days (healthy volunteers) | | May continue through conditioning | |
| 3 | Crizotinib  (PO)  (42, 43) | - Hepatically metabolized via CYP3A4/5 followed by conjugation to inactive metabolites  - Excreted unchanged in the feces and urine  - Inhibitor of CYP3A4 (moderate), CYP2B6, OCT1, OCT2  - Substrate of CYP3A4 (strong)  - P-gp/ABCB1 substrate | PK:  Busulfan  Cyclophosphamide  Etoposide  Melphalan  Thiotepa  PD:  Hepatotoxicity  Pulmonary toxicity  GI toxicity | Children:  4.1-13.1 hr  Adult: 42 hr | | 7 days | 7 days |
| 2 | Cyclosporine  (IV, PO)  (44, 45) | - Hepatically metabolized by CYP3A4/5 to inactive metabolites  - Excreted primarily in the bile as inactive metabolites  - Undergoes hepatic recirculation  - Inhibitor of CYP2C9 (weak), CYP3A4 (weak)  - Substrate of CYP3A4 (strong)  - P-gp/ABCB1 inhibition and substrate | PK:  Busulfan  Cyclophosphamide  Etoposide  Melphalan  Thiotepa  PD:  Renal toxicity  Thrombotic microangiopathy  Neurotoxicity  Hepatotoxicity | Children: 9.3 hr  Adult: 10-27 hr | | 3 days | May continue through conditioning |
| 3 | Cytarabine  (IV, IT)  (46, 47) | - Hepatically metabolized via nucleotide kinases to active and inactive metabolites  - IT undergoes minimal conversion to inactive metabolite  - Excreted in the urine as inactive metabolite  - Not a CYP inducer or inhibitor | PK: None  PD: Myelosuppression | Children:  Initial: 10 minutes Terminal: 4 hr  Adult:  Terminal: 1-3 hr (plasma)  2 hr (cerebrospinal fluid) | | 2 days (IV) | 1 day (IV) |
|  |  |  |  |  |  | No IT cytarabine within 7 days prior to conditioning or radiation | |
| 1 | Dapsone  (PO)  (48, 49) | - Hepatic metabolism via acetylation and hydroxylation followed by glucuronide conjugation to metabolites  - Excreted in the urine as active drug and metabolites  - Not a CYP inducer or inhibitor  - Weak substrate of CYP2C19, CYP2C8, CYP2C9, CYP2E1  - Substrate of CYP3A4 (strong) | PK: None  PD: Hepatotoxicity  Hematologic toxicity  methemoglobinemia | Children: 15.1 hr  Adult: 28 hr  (10-50 hr) | | 1 day | May continue through conditioning |
| 2 | Daratumumab  (IV)  (50) | - Metabolism unknown; Likely via degradation into small peptides and amino acids via catabolic pathways  -Excretion unknown  - Not a CYP inducer or inhibitor | PK: None  PD: Neutropenia, thrombocytopenia | Children: Unknown  Adult: 18 hr  (9-27 hr) | | May continue through conditioning | |
| 3 | Dasatinib  (PO)  (42, 51-53) | - Hepatically metabolized via CYP3A4, oxidation and conjugation to active and inactive metabolites  - Excreted in the feces as active and inactive metabolites  - Inhibitor of CYP3A4 (weak)  - Substrate of CYP3A4 (strong) | PK:  Busulfan  Cyclophosphamide  Etoposide  Melphalan  Thiotepa  PD: Myelosuppression | Children: 2-5 hr  Adult: 3-5 hr | | 7 days | 3 days |
| 5 | Deferasirox  (PO)  (54-56) | - Extensive hepatic metabolism primarily via glucuronidation via UGT1A1 and UGT1A3 to active metabolite (with minor pharmacologic impact) and other inactive metabolites  - Undergoes enterohepatic recirculation  - Excreted in the feces (84%), bile and urine (8%) as active drug and metabolites primarily  - Inhibitor of CYP1A2 (moderate), CYP2C8 (moderate)  - Substrate of UGT1A1 | PK: Busulfan  PD:  Renal toxicity  Hepatotoxicity  GI toxicity  Myelosuppression | Children: Unknown  Children may have reduced exposure compared to adults (up to 50%)  Adult: 8-16 hr | | 3 days | 1 day |
|  |  |  |  |  |  | The more conservative approach is to hold deferasirox for 4 weeks prior to busulfan therapy, if clinically feasible. This is standard practice at certain institutions and is based on 2 case reports demonstrating increased busulfan exposure in the setting of concomitant deferasirox (see citations). | |
| 5 | Deferiprone  (PO)  (57) | - Extensive hepatic metabolism via glucuronidation by UGT1A6 to in active metabolite  - Excreted in the urine as an inactive metabolite  - Not a CYP inducer or inhibitor  - Substrate of UGTs | PK: Busulfan  PD: Agranulocytosis Neutropenia Hepatotoxicity | Children: unknown  Adult: 1-2.5 hr | | 1 day | 1 day |
| 5 | Deferoxamine  (SQ)  (58) | - Metabolized by plasma enzymes to active and inactive metabolites  - Excreted in the urine as unchanged drug and metabolites; Also, feces via bile  - Not a CYP inducer or inhibitor | PK: Busulfan  PD: Renal toxicity Pulmonary toxicity | Children: Unknown  Adult: 1-6 hr | | 1 day | 1 day |
| 2 | Dexamethasone  (IV, IM, PO)  (59-61) | - Hepatically metabolized by CYP3A4 to inactive metabolites  - Excreted in the urine  - Induces CYP3A4 (weak)  - Substrate of CYP3A4 (strong)  - P-gp/ABCB1 substrate | PK:  Busulfan  Cyclophosphamide  Etoposide  Melphalan  Thiotepa  PD:  Thromboembolic risk  Immunosuppression | Children:  <2 year: 2.3-9.5 hr  8-16 year: 2.82-7.5 hr  Adult: 3-6 hr | | 7-14 days: Consult with prescribing provider to develop adequate taper resulting in discontin-uation at least 14 days prior to conditioning | May continue through conditioning |
| 5 | Digoxin  (IV, PO)  (62, 63) | - Metabolized via hydrolysis in the intestines to prior absorption. Once absorbed, metabolized hepatically via glucuronidation and sulfation to active and inactive metabolites  - Excreted in the urine as unchanged drug and metabolites  - Substrate of CYP3A4 (weak)  - P-gp substrate  - Not a CYP inducer or inhibitor | PK: None  PD: None | Neonates:  Premature: 61–170 hr  Full-term: 35–45 hr  Infant: 18–25 hr  Children: 18–36 hr  Adult: 36–45 hr | | May continue through conditioning however conditioning agents can lead to subtherapeutic digoxin levels | |
| 5 | Diphenhydramine  (IV, PO)  (64) | - Metabolized in the intestines prior to absorption. Once absorbed, hepatically metabolized via n-demethylation to inactive metabolites  - Minor metabolism via lungs and kidneys  - Excreted in the urine as metabolites and unchanged drug  - Inhibitor of CYP2D6 (weak)  - Weak substrate of CYPs CYP1A2, CYP2C19, CYP2C9, CYP2D6 | PK: None  PD: None | Children: 5 hr  (4-7 hr)  Adult: 9 hr  (7-12 hr) | | May continue through conditioning | |
| 5 | Dronabinol  (PO)  (65, 66) | - Metabolized in the intestines to active metabolites prior to absorption. Once absorbed, hepatically metabolized via hydroxylation and oxidation by CYP2C9 and 3A4 to active and inactive metabolites  - Excreted in the feces (50%) and urine (10%) mainly as inactive metabolites  - Substrate of CYP2C9 (strong), CYP3A4 (weak)  - Not a CYP inducer or inhibitor | PK: None  PD: Neurotoxicity | Children: unknown  Adult: Biphasic  Alpha: 0.5-4 hr  Beta: 25-36 hr | | May continue through conditioning | |
| 4 | Duloxetine  (PO)  (67, 68)  *(see alternative options in*  *Table 2)* | - Hepatically metabolized by CYP1A2 and CYP2D6 to inactive metabolites  - Excreted in the urine (70%) as inactive metabolites and feces (20%) as drug and inactive metabolites  - Inhibitor of CYP2D6 (moderate)  - Strong substrate of CYPs CYP1A2 & CYP2D6 | PK:  Busulfan  Cyclophosphamide  Etoposide  Melphalan  Thiotepa  PD: None | Children: 10.4 hr  Adult: 12 hr  (8-17 hr) | | Consult with prescribing provider to develop adequate taper resulting in discontinuation at least 7 days prior to conditioning | |
| 5 | Eltrombopag  (PO)  (69, 70) | - Hepatically metabolized via oxidation by CYP1A2 & 2C8, and via glucuronidation by UGT1A1 & UGT1A3 to inactive metabolites  - Excreted in the feces as unchanged drug and urine as inactive metabolites  - Inhibitor of UGT1A3, UGT1A6, UGT2B15, and UGT2B7  - In vitro inhibition CYP2C8, CYP2C9, OATP1B1  - Substrate of CYP1A2 (weak), CYP2C8 (weak), UGT1A1, UGT1A3 | PK:  Busulfan  Cyclophosphamide  Etoposide  Melphalan  Thiotepa  PD:  Hepatotoxicity Thromboembolic events | Children: Unknown  Adult: 21-35 hr | | 14 days | 7 days |
| 2 | Emapalumab-izsg  (IV)  (71) | - Metabolized into small peptides and amino acids via protein catabolic pathways  - Not a CYP inducer or inhibitor | PK: None  PD: None | Children: Unknown  Adult:  2.5-18.9 days | | May continue through conditioning | |
| 5 | Enoxaparin  (IV, SQ)  (72) | - Hepatically metabolized via desulfation to inactive or mildly active metabolites  - Excreted via renal elimination as active and inactive fragments  - Not a CYP inducer or inhibitor | PK: None  PD: Thrombocytopenia | Children: Unknown  Adult: 4.5-7 hr | | May continue through conditioning | |
| 3 | Etoposide  (IV, PO)  (73, 74) | - Hepatically metabolized via O-demethylation by CYP3A4/5 and glutathione/glucuronide conjugation by GSTT1/GSTP1 and UGT1A1 to active and inactive metabolites  - Excreted in the urine (55%), bile and the feces (44%) as drug and inactive metabolites  - Substrate of CYP3A4 (strong), CYP1A2 (weak), CYP2E1 (weak)  - P-gp/ABCB1 substrate  - Not a CYP inducer or inhibitor | PK:  Busulfan  Cyclophosphamide  Melphalan  Thiotepa  PD: Myelosuppression | Children: Biphasic  Alpha :0.82 hr  Beta: 6.5 hr  Adult: 6-7 hr | | 2 days | 2 days |
| 1 | Fluconazole  (IV, PO)  (75, 76) | - Minimal hepatic metabolism to inactive metabolites  - Excreted in the urine primarily as active drug  - Inhibitor of CYP2C19 (strong), CYP2C9 (moderate), CYP3A4 (moderate)  - P-gp inhibition | PK:  Busulfan  Cyclophosphamide  Etoposide  Melphalan  Thiotepa  PD: Hepatotoxicity | Children:  0-6 days: 88.6 hr  7-13 days: 67.5 hr  14 days: 55.2 hr  >2 weeks: 20 hr  Adult: 30 hr  (20-50 hr) | | 7 days | May continue through conditioning |
| 4 | Fluoxetine  (PO)  (77)  *(see alternative options in*  *Table 2)* | - Hepatically metabolized via demethylation by CYP isoenzymes, notably CYP2C19 and 2D6 to active metabolite. Subsequently metabolized via glucuronidation  - Excreted in the urine  - Inhibitor of CYP2C19 (moderate), CYP2D6 (strong)  - Weak substrate of CYPs CYP1A2, CYP2B6, CYP2C19, CYP2C9, CYP2D6, CYP2E1, & CYP3A4 | PK:  Busulfan  Cyclophosphamide  Etoposide  Melphalan  Thiotepa  PD: None | Children: Unknown  Adult:4-6 days  with chronic administration  4-16 days for active metabolite | | Consult with prescribing provider to develop plan for discontinuation at least 2 months prior to conditioning | |
| 4 | Fluvoxamine  (PO)  (78)  *(see alternative options in*  *Table 2)* | - Extensive hepatic metabolism via oxidative deamination and demethylation to inactive metabolites  - Excreted in the urine as inactive metabolites  - Inhibitor of CYP1A2 (strong), CYP2C19 (moderate), CYP2C9 (weak), CYP2D6 (weak), CYP3A4 (weak)  - Weak substrate of CYP1A2 & CYP2D6 | PK:  Busulfan  Cyclophosphamide  Etoposide  Melphalan  Thiotepa  PD: None | Children: Unknown  Adult: ~14-16 hr | | Consult with prescribing provider to develop adequate taper resulting in discontinuation at least 7 days prior to conditioning | |
| 5 | Fosaprepitant  (IV)  (79) | - Prodrug of aprepitant  - Extensive hepatic metabolism via CYP3A4, CYP1A2 and CYP2C19 to weakly active metabolites  - Excreted via metabolism  - Inhibitor of CYP3A4 (moderate)  - Inducer of CYP2C9 (weak) with prolonged use | PK:  Busulfan  Cyclophosphamide  Etoposide  Melphalan  Thiotepa  PD: None | Children: 2-5 hr  Adult: 9-13 hr | | If consistent use for > 1 week, 14 days | If minimal/ inconsistent use, 2 days |
| 5 | Fosphenytoin  (IV)  (80) | - Prodrug converted to active form phenytoin via hydrolysis  - Phenytoin is hepatically metabolized by CYP2C9 and 2C19 to metabolites  - Excreted in urine as inactive metabolites  - Inducer of CYP1A2 (weak), CYP2B6 (weak), CYP3A4 (strong), UGT1A1, UGT1A4  -Substrate of CYP2C19 (strong), CYP2C9 (strong) CYP3A4 (weak)  - P-gp/ABCB1 inducer | PK:  Busulfan  Etoposide  Thiotepa  PD: None | Children & Adult (phenytoin): 12-29 hr | | Consult with prescribing provider to develop plan for discontinuation at least 14 days months prior to conditioning | |
| 5 | Gabapentin  (PO)  (81, 82) | - Not metabolized  - Eliminated via renal excretion as unchanged parent drug  - Not a CYP inducer or inhibitor | PK: None  PD: None | Children: 4 hr  Adult: 5-7 hr | | May continue through conditioning | |
| 1 | Ganciclovir  (IV)  (83) | - Little to no metabolism  - Eliminated via renal excretion as unchanged parent drug  - Not a CYP inducer or inhibitor | PK: None  PD: Myelosuppression Renal toxicity | Children:  2-49 days: 2.4 hr  9 month-12 years: 2.4 hr (1.7-3.1 hr)  Adult: 3.5 hr (2.6-4.4 hr) | | May continue through conditioning | |
| 3 | Gemtuzumab ozogamicin  (IV)  (84-87) | - Drug conjugate calicheamicin derivative is metabolized through nonenzymatic reduction  - Not a CYP inducer or inhibitor | PK: None  PD: Busulfan  Cyclophosphamide  Etoposide  Melphalan  Thiotepa  Associated with an increased risk of veno-occlusive disease | Children: 65 hr  Adult:  62 hr after first dose  90 hr after second dose | | ≥ 2 months from last dose of gemtuzumab ozogamicin to HCT | |
| 3 | Gilteritinib  (PO)  (88, 89) | - Hepatically metabolized via N-de-alkylation and oxidation by CYP3A4 to inactive metabolites  - Excreted in feces (64.5%) and urine (16.4%) as unchanged parent drug and metabolites  - Substrate of CYP3A4 (strong)  - P-gp/ABCB1 substrate (weak) | PK:  Busulfan  Cyclophosphamide  Etoposide  Thiotepa  PD: None | Children: Unknown  Adult: 113 hr | | 14 days | 7 days |
| 5 | Glutamine  (PO)  (90) | - Hepatically metabolized to glutamate & ammonia  - Not a CYP inducer or inhibitor | PK: None  PD: None | Children: Unknown  Adult: 1 hr | | May continue through conditioning | |
| 5 | Granisetron  (IV, PO, SQ)  (91) | - Hepatically metabolized via N-demethylation by CYP1A1 and CYP3A4, oxidation and conjugation to potentially partially active metabolites  - Excreted in the urine (48%) and feces (38%) as metabolites and unchanged drug  - Substrate of CYP3A4 (weak), CYP1A1 (weak)  - Not a CYP inducer or inhibitor | PK: None  PD: None | Children: Unknown  Adult:  Oral: 6 hr  IV: 5-9 hr  SQ: ~24 hr | | May continue through conditioning | |
| 2 | Hydrocortisone  (IV, IM, PO, SQ)  (92) | - Hepatically metabolized by CYP3A4 and by glucuronidation to inactive metabolites  - Excreted in the urine  - Substrate of CYP3A4 (weak)  - Not a CYP inducer or inhibitor | PK: None  PD:  Immunosuppression | Children & Adult:  PO/IV: 1.5-2 hr  IM: 2.2 hr  SQ: 4.7 hr | | May continue through conditioning | |
| 3 | Hydroxyurea  (PO)  (93-95) | - Significantly metabolized by intestinal bacteria and hepatically metabolized to inactive metabolites  - Excreted in the urine  - Not a CYP inducer or inhibitor | PK: None  PD: Myelosuppression | Children: 1.7 hr  (0.65-3 hr)  Adult: 2-4.5 hr | | 7 days | 1 day |
| 3 | Imatinib  (PO)  (96) | - Hepatically metabolized mainly by CYP3A4 (and other CYP450s) to active metabolite  - Excreted in the feces and urine as active metabolites and unchanged drug  - Inhibitor of CYP3A4 (moderate)  - Substrate of CYP3A4 (strong)  - Weak substrate of CYP1A2, CYP2C19, CYP2C8, CYP2C9, CYP2D6  - P-gp/ABCB1 substrate  - hENT inhibitor | PK:  Busulfan  Cyclophosphamide  Etoposide  Melphalan  Thiotepa  Fludarabine  PD: Myelosuppression Hepatotoxicity Renal toxicity | Children: 14.8 hr  Adult: 18 hr  (Active metabolite: 40 hr) | | 7 days | 3 days |
| 3 | Inotuzumab Ozogamicin  (IV)  (97-100) | - Drug conjugate N-acetyl-gamma-calicheamicin dimethylhydrazine is metabolized via nonenzymatic reduction  - Not a CYP inducer or inhibitor | PK: None  PD:  Busulfan  Cyclophosphamide  Etoposide  Melphalan  Thiotepa  Associated with increased risk of veno-occlusive disease | Children: Unknown  Adult: 12.3 days | | ≥ 3 months from last dose of Inotuzumab to HCT if possible (recommendation extrapolated from gemtuzumab data since there is not clear guidance on this for Inotuzumab)  For individuals receiving Inotuzumab proceeding to HCT, limit Inotuzumab to 2 cycles | |
| 1 | Isavuconazole  (IV, PO)  (101, 102) | - Prodrug converted to active form in blood by esterases  - Hepatically metabolized by CYP3A4/5 and UGTs to inactive metabolites  - Excreted primarily in the feces  - Inhibitor of CYP3A4 (moderate) and OCT2  - Inducer of CYP2B6 (weak)  - Substrate of CYP3A4 (strong) | PK:  Busulfan  Cyclophosphamide  Etoposide  Melphalan  Thiotepa  PD: Hepatotoxicity | Children: Unknown  Adult: 130 hr | | 14 days | 7 days |
| 3 | Isotretinoin  (PO)  (103) | - Hepatically metabolized by CYP2B6, 2C8, 2C9 and 3A4 to active metabolite  - Excreted in the urine and feces as active drug  - Substrate of CYP2B6, CYP2C8, CYP2C9, and CYP3A4 | PK: None  PD: None | Adolescent & Adult: 21 hr  (13-29 hr) | | 7 days | 3 days |
| 1 | Itraconazole  (PO)  (104, 105) | - Hepatically metabolized via oxidation by CYP3A4 to active and inactive metabolites  - Excreted in the urine as inactive metabolites and feces as active metabolites and parent drug  - Inhibitor of CYP3A4 (strong)  - Substrate of CYP3A4 (strong)  - P-gp/ABCB1 inhibition | PK:  Busulfan  Cyclophosphamide  Etoposide  Melphalan  Thiotepa  PD: Hepatotoxicity | Infant and Children  Oral solution:  6 month-2year: 47 hr  2-5 year.: 5.3-55.9 hr  5-12 year.: 18.7-37.9 hr  Adult  Single dose: 16-28 hr  Multiple doses: 34-42 hr | | 10 days | 7 days |
| 5 | Ketamine  (IV, PO)  (106) | - Hepatically metabolized via N-dealkylation, hydroxylation, and conjugation to inactive or weakly-active metabolites  - Excreted in the urine (91%)  - Substrate of CYP2B6 (strong), CYP2C9 (weak), CYP3A4 (weak)  - Not a CYP inducer or inhibitor | PK: None  PD: None | Children & Adult: 0.75 hr | | May continue through conditioning | |
| 5 | Laronidase  (IV)  (107) | - Not fully elucidated. Presumed nonspecific peptide hydrolysis  - Excretion unknown  - Not a CYP inducer or inhibitor | PK: None  PD: None | Children:  6 month-5 years:  0.3-1.9 hr  Children >6 years & Adult: 1.5-3.6 hr | | May continue through conditioning | |
| 1 | Levofloxacin  (IV, PO)  (108-110) | - Limited metabolism  - Excreted in the urine as unchanged parent drug.  - Potential CYP2C9 inhibition  - Well documented increase in warfarin due to inhibition of CYP2C9 leading to potential interactions with conditioning agents | PK:  Busulfan  Cyclophosphamide  Etoposide  Melphalan  Thiotepa  PD: None | Children:  6 month-5 years: 4 hr  5 to 10 years: 4.8 hr  10 to 16 years:5-6 hr  Adult: 3-8 hr | | 1 day | May continue through conditioning |
| 5 | Lorazepam  (IV, PO)  (111, 112) | - Hepatically metabolized via glucuronidation to inactive metabolite  - Excreted in the urine (88%) as inactive metabolite and feces (7%)  - Not a CYP inducer or inhibitor | PK: None  PD: None | Neonate:  IV: 40 hr  (18–73 hr)  Children:  5 month-3 years (IV): 15.8 hr  (5.9-28.4 hr)  3-12.9 years (IV): 16.9 hr  (7.5 -40.6 hr)  13-<18 years (IV): 17.8 hr  (8.2-42 hr)  Adult: PO 12 hr  IV 14 hr  IM 13-18 hr | | May continue through conditioning | |
| 5 | Megestrol  (PO)  (113, 114) | - Hepatically metabolized via glucuronidation to inactive metabolites  - Excreted in the urine and feces  - Substrate of CYP3A4/5 and UGT2B17 | PK: None  PD: None | Children: Unknown  Adult: 34 hr  (13-105 hr) | | 1 day | 1 day |
| 1 | Meropenem  (IV)  (115-117) | - Hepatically metabolized via hydrolysis to inactive metabolite  - Excreted in the urine as unchanged parent drug (70%) and inactive metabolite (28%)  - Nonrenal metabolism can increase up to 50% in patients with CrCL <20 ml/min  - Not a CYP inducer or inhibitor | PK: None  PD: None | Children:  <3 month: 2.7 hr (1.6-3.8 hr)  3 month -1.9 years: 1.5 hr  >2-year-old to Adult: 1hr | | May continue through conditioning | |
| 5 | Methadone  (IV, PO)  (118) | - Hepatically metabolized via N-demethylation by the CYP450 isoenzymes to inactive metabolites  - Excreted in the urine and feces  - Inhibitor of CYP2D6 (weak)  - Substrate of CYP2B6 (strong), CYP3A4 (strong), CYP2C19 (weak), CYP2C9 (weak), CYP2D6 (weak) | PK: Thiotepa  PD: None | Children and Adolescent: 19.2 hr  (5.6-62 hr)  Adult: 8-59 hr  Significant pharmacogenomic effects may result in variable plasma levels and rates of elimination | | Consult with prescribing provider to develop adequate taper resulting in discontinua-tion at least 14 days prior to conditioning | May continue through conditioning |
| 2 | Methotrexate  (IV, IT, PO)  (2, 119-121) | - Partial metabolism by intestinal bacteria via carboxypeptidase. Hepatically metabolized by aldehyde oxidase to active metabolites  - Eliminated via renal excretion as unchanged parent drug (80 – 90%)  - Not a CYP inducer or inhibitor  - Substrate of PgP/ABCB1 (weak) | PK: None  PD: Myelosuppression | Children: 0.7-5.8 hr  Adult: 3-15 hr | | 14 days | 7 days |
|  |  |  |  |  |  | No IT methotrexate within 14 days prior to start of conditioning or radiation | |
| 5 | Metoclopramide  (IV, PO)  (122) | - Hepatically metabolized via oxidation, glucuronidation and sulfate conjugation to inactive metabolite  - Excreted in the urine  - Substrate of CYP2D6 (strong), CYP1A3 (weak)  - Not a CYP inducer or inhibitor | PK: None  PD: None | Neonate: 5.4 hr  Infant: 4.15 hr  Children: 4 hr  Adult: 5-6 hr | | May continue through conditioning | |
| 1 | Metronidazole  (IV, PO)  (123-126) | - Hepatically metabolized via hydroxylation, oxidation, and glucuronidation to active and inactive metabolites  - Excreted in the urine as unchanged drug (20%) and metabolites (60-80%), feces (6-15%)  - Inhibitor of CYP2C9 (weak)  - Substrate of CYP2A6  - Down-regulates expression of CYP2C8, CYP2C9, and CYP3A4 | PK:  Busulfan  Cyclophosphamide  Etoposide  Melphalan  Thiotepa  PD: None | Children & Adolescent:  6-10 hr  Adult: ~8 hr | | 7 days | 3 days |
| 3 | Midostaurin  (PO)  (127-129) | - Hepatically metabolized via CYP3A4 to active metabolites  - Excreted in the feces (95%) as parent drug and active metabolites and urine (5%)  - Substrate of CYP3A4 (major)  - Auto-inducer of CYP3A4  - Inhibits OATP1A1/SLCO1A1  - Induces MRP2 | PK:  Clofarabine  Cyclophosphamide  Etoposide  Thiotepa  PD: Myelosuppression | Children: unknown  Adult:  Parent drug: 19 hr  Metabolites:  32 – 482 hr | | 14 days | 7 days |
| 1 | Micafungin  (IV)  (130-132) | - Hepatically metabolized into multiple metabolites  - Excreted in the feces  - Substrate of CYP3A4 (weak) | PK: None  PD: None | Children:  Premature & term neonates, infant <4 months: 11-13.6 hr  4 months–16 year: 10-21 hr  Adult: 10-17 hr | | May continue through conditioning | |
| 5 | Milrinone  (IV)  (133) | - Hepatically metabolized to glucuronide conjugate (minor); majority is not metabolized  - Excreted in the urine as unchanged drug (major)  - Not a CYP inducer or inhibitor | PK: None  PD: None | Infant & Children: 2 hr (1-5 hr)  Adult: 2.4 hr | | May continue through conditioning | |
| 3 | Nilotinib  (PO)  (134) | - Hepatically metabolized via oxidation and hydroxylation by CYP3A4 to inactive metabolites  - Inhibitor of CYP3A4 (moderate)  - Substrate of CYP3A4 (major)  - P-gp /ABCB1 substrate | PK:  Busulfan  Cyclophosphamide  Etoposide  Melphalan  Thiotepa  PD: Myelosuppression  Hepatotoxicity | Children: unknown  Adult: 17 hr | | 7 days | 5 days |
| 3 | Nitazoxanide  (PO)  (135) | - Prodrug converted to active form tizoxanide via hepatic metabolism  - Not a CYP inducer or inhibitor | PK: None  PD: None | Children: unknown  Adult: 1-1.6 hr | | May continue through conditioning | |
| 5 | Olanzapine  (IM, PO)  (136, 137) | - Hepatically metabolized via oxidation and glucuronidation by CYP1A2 and 2D6  - Excreted in the urine (57%) and feces (30%) as inactive metabolites  - Substrate of CYP1A2 (strong), CYP2D6 (weak) UGT1A4  - Not a CYP inducer or inhibitor | PK: None  PD: None | Children & Adolescent:  10-18 year: 32-42 hr  Adult: 30 hr  (21-54 hr) | | May continue through conditioning | |
| 5 | Ondansetron  (IV, PO)  (138) | - Hepatically metabolized via hydroxylation followed by conjugation  - Excreted in the urine and feces  - Weak substrate of CYPs CYP1A2, CYP2C9, CYP2D6, CYP2E1 and CYP3A4  - P-gp/ABCB1 substrate  - Not a CYP inducer or inhibitor | PK: None  PD: None | Children & Adolescent with cancer:  4-18 year: 2 hr  Adult: 3-6 hr  (mild hepatic impairment 12 hr,  moderate hepatic impairment: 20 hr) | | May continue through conditioning | |
| 5 | Oxcarbazepine  (PO)  (139) | - Prodrug converted to its active form  - Active form is hepatically metabolized via glucuronidation  - Excreted in the urine  - Inducer of CYP3A4 (weak) | PK:  Busulfan  Cyclophosphamide  Etoposide  Melphalan  Thiotepa  PD: None | Children:  2-5 years: 4.8-6.7 hr  6-12 years: 7.2-9.3 hr  Adult: 9 hr  (7-11 hr) | | Consult with prescribing provider to develop adequate taper resulting in discontinuation at least 14 days prior to conditioning | |
| 5 | Pantoprazole  (IV, PO)  (140) | - Hepatically metabolized via demethylation by CYP2C19 to inactive metabolites  - Excreted in the urine and feces  - Weak substrate of CYP2C19, CYP2D6 and CYP3A4  - Not a CYP inducer or inhibitor | PK: None  PD: None | Neonate: 3 hr  Children to Adult: 1 hr | | May continue through conditioning | |
| 4 | Paroxetine  (PO)  (141, 142)  *(see alternative options in*  *Table 2)* | - Hepatically metabolize via oxidation and methylation followed by conjugation to mainly inactive metabolites  - Inhibitor of CYP2D6 (strong)  - Substrate of CYP2D6 (strong) | PK:  Busulfan  Cyclophosphamide  Etoposide  Melphalan  Thiotepa  PD: None | Children:  5.9-16.3 hr  Adult: 15-21 hr | | Consult with prescribing provider to develop adequate taper resulting in discontinuation at least 7 days prior to conditioning | |
| 1 | Pentamidine  (IV, INH)  (143, 144) | - Hepatically metabolized via CYP system with no inhibitory effects  - Excreted in the urine  - Not a CYP inducer or inhibitor | PK: None  PD: None | Children: unknown  Adult (IV): 5-7 hr | | May continue through conditioning | |
| 5 | Phenytoin  (PO)  (145) | - Hepatically metabolized via oxidation and glucuronide conjugation  - Undergoes enterohepatic recirculation  - Excreted in the urine  - Inducer of CYP1A2 (weak), CYP2B6 (weak), CYP3A4 (strong), UGT1A1, UGT1A4  - Strong substrate of CYP2C19, CYP2C9, CYP3A4  - P-gp/ABCB1 inducer | PK:  Busulfan  Cyclophosphamide  Etoposide  Melphalan  Thiotepa  PD: None | Children: unknown  Adult: 7-42 hr | | Consult with prescribing provider to develop adequate taper resulting in discontinuation at least 14 days prior to conditioning | |
| 1 | Piperacillin-tazobactam  (IV)  (146) | - Eliminated via renal excretion primarily as unchanged drug  -Excreted in the urine  - Not a CYP inducer or inhibitor | PK: None  PD: Renal toxicity | Neonate: 3.5-14 hr  1-6 month.: 47 min  Children & Adult: 1-2 hr | | May continue through conditioning | |
| 3 | Ponatinib  (PO)  (147, 148) | - Hepatically metabolized by CYP3A4, CYP2C8, CYP2D6, CYP3A5 to inactive metabolites  - Excreted in the feces (87%) and urine (5%)  - Substrate of CYP3A4 (strong), CYP2D6 (weak)  - P-gp/ABCB1 substrate | PK:  Busulfan  Cyclophosphamide  Etoposide  Melphalan  Thiotepa  PD: Myelosuppression Hepatotoxicity | Children: unknown  Adult: 24 hr | | 7 days | 5 days |
| 1 | Posaconazole  (IV, PO)  (149, 150) | - Minimal hepatic metabolism via glucuronidation into inactive metabolites  - Excreted in the feces as unchanged parent drug  - Inhibitor of CYP3A4 (strong)  - Substrate of UGT1A4 | PK:  Busulfan  Cyclophosphamide  Etoposide  Melphalan  Thiotepa  PD: Hepatotoxicity | Children: 20-66 hr  Adult: 26- 35 hr | | 10 days | 7 days |
| 5 | Pravastatin  (PO)  (151, 152) | - Hepatically metabolize via hydroxylation to minimally active metabolite  - Excreted in the feces (70%) and urine (20%)  - Substrate of CYP3A4 (weak)  - Not a CYP inducer or inhibitor | PK: None  PD: None | Children:  5-15.6 year: 1.6 hr  (0.85-4.2 hr)  Adult: 2-3 hr | | May continue through conditioning | |
| 2 | Prednisolone  (PO)  (153) | - Hepatically metabolized to  inactive metabolize  - Excreted in the urine  - Substrate of CYP3A4 (weak)  - P-gp/ABCB1 inducer | PK: Busulfan  Cyclophosphamide  Etoposide  Melphalan  Thiotepa  PD:  Immunosuppression | Children: 2.2 hr  Adult: 2-3 hr | | 7-14 days: Consult with prescribing provider to develop adequate taper resulting in discontin-uation at least 14 days prior to conditioning | May continue through conditioning |
| 2 | Prednisone  (PO)  (59, 154, 155) | - Hepatically metabolized to active metabolite  - Excreted in the urine  - Inducer of CYP3A (weak)  - Substrate CYP3A4 (weak)  - P-gp/ABCB1 inducer | PK:  Busulfan  Cyclophosphamide  Etoposide  Melphalan  Thiotepa  PD:  Immunosuppression | Children: 18-36 hr  Adult: 2-3 hr | | 7-14 days: Consult with prescribing provider to develop adequate taper resulting in discontin-uation at least 14 days prior to conditioning | May continue through conditioning |
| 5 | Pregabalin  (PO)  (156) | - Excreted via renal elimination as unchanged drug  - Not a CYP inducer or inhibitor | PK: None  PD: None | Children:  <6-year-old: 3-4 hr  7 to 16-year-old: 4-6 hr  Adult: 6.3 hr | | May continue through conditioning | |
| 1 | Rifabutin  (PO)  (157) | - Hepatically metabolized to active and inactive metabolites  - Excreted in the urine (53%) and feces (30%)  - Inducer of CYP3A4 (moderate), CYP2C9 (weak), UGT1A4  - Substrate of CYP3A4 (major), CYP1A2 (weak) | PK:  Busulfan  Cyclophosphamide  Etoposide  Melphalan  Thiotepa  PD: Hematologic toxicity (rare) | Children: unknown  Adult: 45 hr  (16-69 hr) | | 14 days | May continue through conditioning |
| 1 | Rifampin  (PO)  (158) | - Hepatically metabolized  - Undergoes enterohepatic recirculation  - Excreted in the feces (60-65%) and urine (30%) as unchanged drug  - Inducer of CYP3A4 (strong), CYP2C19 (strong), CYP2C8 (moderate), CYP2C9 (moderate), CYP2B6 (moderate), CYP1A2 (weak), UGT1A1, UGT1A9  - Inhibitor of OATP1B1/1B3  - Substrate of OATP1B1/1B3  - P-gp/ABCB1 substrate and inducer | PK:  Busulfan  Cyclophosphamide  Etoposide  Melphalan  Thiotepa  PD:  Hepatotoxicity  Hematologic toxicity | Children: 1-4 hr  Adult: 1.5-5 hr | | 14 days  Consult with prescribing provider to develop adequate plan for discontinuation at least 14 days prior to conditioning | |
| 5 | Scopolamine  (transdermal)  (159) | - Hepatically metabolized primarily to inactive metabolites  - Excreted in the urine  - Not a CYP inducer or inhibitor | PK: None  PD: None | Transdermal patch: 6-8 hr | | May continue through conditioning | |
| 4 | Sertraline  (PO)  (160-162)  *(see alternative options in*  *Table 2)* | - Hepatically metabolized via N-demethylation to partially active and inactive metabolites  - Excreted in the urine (45%) and feces (45%)  - Weak inhibitor of CYP2D6  - Weak substrate of CYP2B6, CYP2C19, CY2C9, CYP2C9, CYP2D6 and CYP3A4 | PK:  Busulfan  Cyclophosphamide  Etoposide  Melphalan  Thiotepa  PD: None | Children:  6-12 year: 26.2 hr  13-17 year: 27.8 hr  Adult: 27.2 hr | | Consult with prescribing provider to develop adequate taper resulting in discontinuation at least 10 days prior to conditioning | |
| 2 | Sirolimus  (PO)  (163, 164) | - Extensive; intestinal wall via P-gp and hepatic metabolism by CYP3A4 inactive metabolites  - Excreted in the feces (91%)  - Substrate of CYP3A4 (major)  - P-gp/ABCB1 substrate | PK:  Busulfan  Cyclophosphamide  Etoposide  Melphalan  Thiotepa  PD: Immunosuppression renal toxicity | Children: 24.5-71 hr  Adult: 61-72 hr | | 7 days | May continue through conditioning |
| 3 | Sorafenib  (PO)  (165, 166) | - Hepatically metabolized via oxidation by CYP3A4 to active metabolite and glucuronidation by UGT1A9  - Excreted in the feces (77%) and urine (19%) as unchanged drug  - Inhibitor of CYP2B6, UGT1A1  - Substrate of CYP3A4 (weak), UGT1A9 | PK:  Busulfan  Cyclophosphamide  Etoposide  Melphalan  Thiotepa  PD: Hepatotoxicity | Children: ≥24 hr but could not be estimated  Adult: 25-48 hr | | 14 days | 7 days |
| 1 | Sulfamethoxazole/Trimethoprim  (IV, PO)  (167) | - Hepatically metabolized via hydroxylation, oxidation, and conjugation  - Excreted in the urine as unchanged parent drug and metabolites  - Inhibitor of CYP2C8 (weak)  - Weak substrate of CYP2C9 & CYP3A4 | PK: None  PD: Myelosuppression | Trimethoprim:  Newborn: ~19 hr  >2 month-1 year: ~4.6 hr  Children:  1-10 year: 3.7-5.5 hr  >10 year: 8.19 hr  Sulfamethoxazole: 9-12 hr, prolonged in renal failure | | Day of admission or 7 days | May continue through conditioning |
| 2 | Tacrolimus  (IV, PO)  (168, 169) | - Hepatically metabolized by CYP3A4/5 to active and inactive metabolites  - Excreted in the bile and feces (93%)  - Substrate of CYP3A4 (strong)  - P-gp/ABCB1 substrate  - Inhibitor of CYP3A4 though no clinically significant effect | PK:  Busulfan  Cyclophosphamide  Etoposide  Melphalan  Thiotepa  PD:  Renal toxicity Neurotoxicity  GI toxicity | Children: 12 hr  Adult: 8.7-37.9 hr | | 3 days | May continue through conditioning |
| 3 | Trametinib  (PO)  (170-172) | - Hepatically metabolized via deacetylation followed by glucuronidation  - Excreted in the feces (>80%) and urine (< 20%) as inactive metabolites  - Inducer of CYP3A4, though no clinically important effect on CYP3A4 sensitive substrate AUC or Cmax  - Inhibitor of CYP2C8 | PK:  Busulfan  Cyclophosphamide  Etoposide  Melphalan  Thiotepa  PD:  Hepatotoxicity  GI toxicity Pulmonary toxicity | Children: unknown  Adult: 4-5 days | | 7 days | 3 days |
| 5 | Ursodiol  (PO)  (173) | - Undergoes hepatic conjugation, biliary excretion and enterohepatic recycling  - Excreted in the feces  - Not a CYP inducer or inhibitor | PK: None  PD: None | Children: unknown  Adult: ~3-6 days | | May continue through conditioning | |
| 1 | Valacyclovir  (PO)  (174, 175) | - Prodrug converted to active form in hepatic first pass  - Acyclovir is minimally metabolized to inactive metabolites  - Excreted in the urine as active drug  - Not a CYP inducer or inhibitor | PK: None  PD:  Myelosuppression | Children: 2.1-2.5 hr  Adult: 2.5-3.3 hr | | May continue through conditioning | |
| 1 | Valganciclovir  (PO)  (176) | - Prodrug converted to active form ganciclovir by intestinal mucosal cells and hepatocytes  - Excreted in the urine as ganciclovir (90%)  - Not a CYP inducer or inhibitor | PK: None  PD: Myelosuppression | Children: 7 hr  (4.2-11 hr)  Adult: 4-7 hr | | May continue through conditioning | |
| 3 | Venetoclax  (PO)  (177) | - Hepatically metabolized by CYP3A4/5 to active metabolite  - Excreted in feces (99.9%) as metabolites and unchanged drug  - Substrate of CYP3A4 (strong)  - P-gp/ABCB1 substrate (weak) | PK: None  PD: Neutropenia, thrombocytopenia, anemia | Children: Unknown  Adult: 26 hr | | 7 days | 3 days |
| 3 | Vincristine  (IV)  (178, 179) | - Hepatically metabolized by CYP3A4 to inactive metabolite  - Excreted in the feces (80%) and urine (10-20%)  - Substrate of CYP3A4 (strong)  - P-gp/ABCB1 substrate | PK: None  PD:  Neurotoxicity hepatotoxicity | Children: 16.7 hr  Adult: 85 hr | | 7 days | 3 days |
| 1 | Voriconazole  (IV, PO)  (180) | -Hepatically metabolized by CYP450s to mainly inactive metabolites  - Inhibitor of CYP3A4 (strong), CYP2C19 (moderate), CYP2C9 (weak)  - Substrate of CYP2C19 (strong), CYP2C9 (weak), CYP3A4 (weak)  - P-gp/ABCB1 inhibitor | PK:  Busulfan  Cyclophosphamide  Etoposide  Melphalan  Thiotepa  PD:  Hepatotoxicity Renal toxicity | Children: 7.5 hr (3.1-21.4 hr)  Adult: 6-9 hr  Significant pharmacogenomic effects may result in variable plasma levels and rates of elimination | | 7 days | 7 days |
| 5 | Voxelotor  (PO)  (181) | - Hepatically metabolized via oxidation mainly by CYP3A4 and to a lesser extent by CYP2C19, CYP2B6, and CYP2C9 and reduction. Followed by glucuronidation  - Excreted in the feces (63%) as unchanged drug and metabolites and in the urine (36%) as unchanged drug  - Weak substrate of CYP3A4, CYP2C19, CYP2B6, CYP2C9  - Inhibitor of CYP3A4 (weak) | PK:  Busulfan  Cyclophosphamide  Etoposide  Melphalan  Thiotepa  PD:  Hepatic impairment | Children: unknown  Adult: 35.5 hr | | 14 days | 7 days |

**Table 2A. Preferred Psychiatric Medications**

These psychiatric medication options that have few drug interactions and might be considered. Keep in mind some of these medications have no IV form so for patients with severe mucositis administration may not be possible. However, many can be crushed, or capsules opened, for mixing with food. Abbreviations: SSRI - Selective Serotonin Reuptake Inhibitor; SNRI - Serotonin-Norepinephrine Reuptake Inhibitor; SIADH - Syndrome of inappropriate antidiuretic hormone secretion

| **Drug**  **(references)** | **Formulations** | **Class** | **Side Effect Profile** | **Recommended Monitoring** | **Clinical Pearls** |
| --- | --- | --- | --- | --- | --- |
| Escitalopram  (182, 183) | Oral solution (5 mg/mL, 1 mg/mL  Oral tablet (5 mg, 10 mg, 20 mg) | SSRI | *Common:*  Nausea & Diarrhea  Insomnia/Agitation  Sexual Disorder  *Other:*  Sweating  Bruxism  SIADH  Increased bleeding risk  Serotonin Syndrome | **Black Box Warning:** Increased risk of suicidal thoughts and behavior in pediatrics (through age 25) | -No IV formulation available  - Taper when discontinuing  - Potential QTc prolongation similar to Citalopram (see below)  - Consider limiting dose to 10 mg/day if concomitant omeprazole |
| Citalopram  (184) | Oral solution (5 mg/mL, 1 mg/mL  Oral tablet (5 mg, 10 mg, 20 mg) | SSRI | *Common:*  Nausea & Diarrhea (Take with food)  Potential Insomnia/Agitation  Sexual disorder  *Other:*  Sweating  Bruxism  SIADH  Increased bleeding risk  Serotonin Syndrome | **Black Box Warning:** Increased risk of suicidal thoughts and behavior in pediatrics (through age 25) | -No IV formulation available  - Taper when discontinuing  - QTc prolongation – FDA warns to avoid if:  (1) pre-existing arrhythmic condition  ***OR***  (2) on CYP 2C19 inhibitors (omeprazole, cimetidine). **Max dose of 20 mg/day of citalopram if on omeprazole** |
| Vilazodone  (185) | Oral tablet (10 mg, 20 mg, 40 mg) | SSRI & Serotonin Agonist | *Common:*  Diarrhea  Nausea  Headache  *Other:*  Seizures (if history present)  Increased bleeding risk  Serotonin Syndrome | **Black Box Warning:** Increased risk of suicidal thoughts and behavior in pediatrics (through age 25) | -No IV formulation available  - Must take with food for adequate absorption |
| Vortioxetine  (186) | Oral tablet (5 mg, 10 mg, 20 mg) | Serotonin Agonist | *Common:*  Sexual disorder  Nausea  Diarrhea  Dizziness  *Other:*  Abnormal bleeding  SIADH  Angle-closure glaucoma  Serotonin Syndrome | **Black Box Warning:** Increased risk of suicidal thoughts and behavior in pediatrics (through age 25) | -No IV formulation available  - May discontinue abruptly unless dose > 15 mg/day  - Max recommended dose in CYP2D6 poor metabolizers is 10 mg/day |
| Mirtazapine  (187) | Oral tablet (7.5 mg, 15 mg, 30 mg, 45 mg) | Tetracyclic Antidepressant | *Common:*  Drowsiness  Dry mouth  Increased appetite  Weight gain  *Other:*  Hyponatremia  Angle-closure glaucoma  Serotonin Syndrome | **Black Box Warning:** Increased risk of suicidal thoughts and behavior in pediatrics (through age 25) | -No IV formulation available  - Potential QTc prolongation  - Taper when discontinuing |
| Venlafaxine  (188) | Oral tablet (25 mg, 37.5 mg, 50 mg, 75 mg, 100 mg)  Oral capsule/tablet extended release (37.5 mg, 75 mg, 150 mg, 225 mg)  Note: **not preferred** cannot be opened/crushed during mucositis | SNRI | *Common:*  Insomnia  Dizziness  Drowsiness  Nausea  Dry mouth  *Other:*  Sexual dysfunction  Elevated blood pressure  Abnormal bleeding  Angle-closure glaucoma  Serotonin Syndrome | **Black Box Warning:** Increased risk of suicidal thoughts and behavior in pediatrics (through age 25) | -No IV formulation available  - Suggested to take with food  - Taper when discontinuing |

**References**

1. Nova Laboratories Ltd., Purixan [package insert]. U.S. Food and Drug Administration website. <https://www.accessdata.fda.gov/drugsatfda_docs/label/2014/205919s000lbl.pdf>. Revised 4/2014. Accessed 5/2021.

2. Balis FM, Holcenberg JS, Poplack DG, Ge J, Sather HN, Murphy RF, et al. Pharmacokinetics and pharmacodynamics of oral methotrexate and mercaptopurine in children with lower risk acute lymphoblastic leukemia: a joint children's cancer group and pediatric oncology branch study. *Blood* (1998) 92(10):3569-77. Epub 1998/11/10. PubMed PMID: 9808549.

3. Lennard L, Davies HA, Lilleyman JS. Is 6-thioguanine more appropriate than 6-mercaptopurine for children with acute lymphoblastic leukaemia? *Br J Cancer* (1993) 68(1):186-90. Epub 1993/07/01. doi: 10.1038/bjc.1993.311. PubMed PMID: 8318412; PubMed Central PMCID: PMCPMC1968314.

4. DSM Pharmaceuticals for GlaxoSmithKline. Tabloid [package insert]. U.S. Food and Drug Administration website. <https://www.accessdata.fda.gov/drugsatfda_docs/label/2004/12429s022lbl.pdf>. Accessed 5/2021.

5. Prescribing information. Busulfex (busulfan). Rockville, MD: Otsuka America Pharmaceutical, Inc., April 2011.

6. Product monograph. Busulfex (busulfan). Saint-Laurent, QC: Otsuka Canada Pharmaceutical Inc., July 2011.

7. Fresenius Kabi USA, LLC. Acetaminophen [package insert]. U.S. Food and Drug Administration website. <https://www.accessdata.fda.gov/drugsatfda_docs/label/2015/204767s000lbl.pdf>. Revised 10/2015. Accessed 5/2021.

8. Almog S, Kurnik D, Shimoni A, Loebstein R, Hassoun E, Gopher A, et al. Linearity and stability of intravenous busulfan pharmacokinetics and the role of glutathione in busulfan elimination. *Biol Blood Marrow Transplant* (2011) 17(1):117-23. Epub 2010/07/06. doi: 10.1016/j.bbmt.2010.06.017. PubMed PMID: 20601034.

9. Nguyen L, Leger F, Lennon S, Puozzo C. Intravenous busulfan in adults prior to haematopoietic stem cell transplantation: a population pharmacokinetic study. *Cancer Chemother Pharmacol* (2006) 57(2):191-8. Epub 2005/09/01. doi: 10.1007/s00280-005-0029-0. PubMed PMID: 16133536.

10. Product Information: acyclovir sodium IV injection, acyclovir sodium IV injection. American Pharmaceutical Partners,Inc, Schaumburg, IL, 2004.

11. Sullender WM, Arvin AM, Diaz PS, Connor JD, Straube R, Dankner W, et al. Pharmacokinetics of acyclovir suspension in infants and children. *Antimicrob Agents Chemother* (1987) 31(11):1722-6. Epub 1987/11/01. doi: 10.1128/aac.31.11.1722. PubMed PMID: 2829714; PubMed Central PMCID: PMCPMC175028.

12. Patheon Mfg Services LLC. Zyloprim, Imuran and Purinethol [package insert]. U.S. Food and Drug Administration website. <https://www.accessdata.fda.gov/drugsatfda_docs/label/2018/016084s044lbl.pdf>. Issued 12/2018. Accessed 5/2021.

13. McGaurn SP, Davis LE, Krawczeniuk MM, Murphy JD, Jacobs ML, Norwood WI, et al. The pharmacokinetics of injectable allopurinol in newborns with the hypoplastic left heart syndrome. *Pediatrics* (1994) 94(6 Pt 1):820-3. Epub 1994/12/01. PubMed PMID: 7970996.

14. Product Information: NORVASC®, amlodipine besylate oral tablets. Pfizer Inc. New York, NY. 2011.

15. Gilead. AmBisome [package insert]. U.S. Food and Drug Administration website. <https://www.accessdata.fda.gov/drugsatfda_docs/label/2008/050740s016lbl.pdf>. Revised 10/2008. Accessed 5/2021].

16. Mehta P, Vinks A, Filipovich A, Vaughn G, Fearing D, Sper C, et al. High-dose weekly AmBisome antifungal prophylaxis in pediatric patients undergoing hematopoietic stem cell transplantation: a pharmacokinetic study. *Biol Blood Marrow Transplant* (2006) 12(2):235-40. Epub 2006/01/31. doi: 10.1016/j.bbmt.2005.10.010. PubMed PMID: 16443521; PubMed Central PMCID: PMCPMC4912056.

17. Merck & Co, Inc. Emend [package insert]. U.S. Food and Drug Administration website. <https://www.accessdata.fda.gov/drugsatfda_docs/label/2015/207865lbl.pdf>. Revised 12/2015. Accessed 5/2017.

18. Chain A, Wrishko R, Vasilinin G, Mouksassi S. Modeling and Simulation Analysis of Aprepitant Pharmacokinetics in Pediatric Patients With Postoperative or Chemotherapy-Induced Nausea and Vomiting. *J Pediatr Pharmacol Ther* (2020) 25(6):528-39. Epub 2020/08/26. doi: 10.5863/1551-6776-25.6.528. PubMed PMID: 32839657; PubMed Central PMCID: PMCPMC7439950.

19. GlaxoSmithKline. Mepron [package insert]. U.S. Food and Drug Administration Website. <https://www.accessdata.fda.gov/drugsatfda_docs/label/2019/020500s017lbl.pdf>. Revised 2/2019. Accessed 5/2021.

20. van de Poll ME, Relling MV, Schuetz EG, Harrison PL, Hughes W, Flynn PM. The effect of atovaquone on etoposide pharmacokinetics in children with acute lymphoblastic leukemia. *Cancer Chemother Pharmacol* (2001) 47(6):467-72. Epub 2001/07/19. doi: 10.1007/s002800000250. PubMed PMID: 11459198.

21. Ben Venue Laboratories for Celegene. Vidaza [package insert]. U.S. Food and Drug Administration website. <https://www.accessdata.fda.gov/drugsatfda_docs/label/2008/050794s011lbl.pdf>. Revised 8/2008. Accessed 5/2021.

22. Van Scoik KG, Johnson CA, Porter WR. The pharmacology and metabolism of the thiopurine drugs 6-mercaptopurine and azathioprine. *Drug Metab Rev* (1985) 16(1-2):157-74. Epub 1985/01/01. doi: 10.3109/03602538508991433. PubMed PMID: 3905317.

23. Product Information: ZITHROMAX®, Azithromycin oral tablets and suspension. Pfizer Inc. New York, NY. 2013.

24. Clements JD, Zhu M, Kuchimanchi M, Terminello B, Doshi S. Population Pharmacokinetics of Blinatumomab in Pediatric and Adult Patients with Hematological Malignancies. *Clin Pharmacokinet* (2020) 59(4):463-74. Epub 2019/11/05. doi: 10.1007/s40262-019-00823-8. PubMed PMID: 31679130; PubMed Central PMCID: PMCPMC7109194.

25. Nagele V, Kratzer A, Zugmaier G, Holland C, Hijazi Y, Topp MS, et al. Changes in clinical laboratory parameters and pharmacodynamic markers in response to blinatumomab treatment of patients with relapsed/refractory ALL. *Exp Hematol Oncol* (2017) 6:14. Epub 2017/05/24. doi: 10.1186/s40164-017-0074-5. PubMed PMID: 28533941; PubMed Central PMCID: PMCPMC5437652.

26. Teachey DT, Rheingold SR, Maude SL, Zugmaier G, Barrett DM, Seif AE, et al. Cytokine release syndrome after blinatumomab treatment related to abnormal macrophage activation and ameliorated with cytokine-directed therapy. *Blood* (2013) 121(26):5154-7. Epub 2013/05/17. doi: 10.1182/blood-2013-02-485623. PubMed PMID: 23678006; PubMed Central PMCID: PMCPMC4123427.

27. Zhu M, Kratzer A, Johnson J, Holland C, Brandl C, Singh I, et al. Blinatumomab Pharmacodynamics and Exposure-Response Relationships in Relapsed/Refractory Acute Lymphoblastic Leukemia. *J Clin Pharmacol* (2018) 58(2):168-79. Epub 2017/09/19. doi: 10.1002/jcph.1006. PubMed PMID: 28922466.

28. Zhu M, Wu B, Brandl C, Johnson J, Wolf A, Chow A, et al. Blinatumomab, a Bispecific T-cell Engager (BiTE((R))) for CD-19 Targeted Cancer Immunotherapy: Clinical Pharmacology and Its Implications. *Clin Pharmacokinet* (2016) 55(10):1271-88. Epub 2016/05/23. doi: 10.1007/s40262-016-0405-4. PubMed PMID: 27209293.

29. Hanley MJ, Mould DR, Taylor TJ, Gupta N, Suryanarayan K, Neuwirth R, et al. Population Pharmacokinetic Analysis of Bortezomib in Pediatric Leukemia Patients: Model-Based Support for Body Surface Area-Based Dosing Over the 2- to 16-Year Age Range. *J Clin Pharmacol* (2017) 57(9):1183-93. Epub 2017/04/19. doi: 10.1002/jcph.906. PubMed PMID: 28419486; PubMed Central PMCID: PMCPMC5561493.

30. Kaygusuz I, Toptas T, Aydin F, Uzay A, Firatli-Tuglular T, Bayik M. Bortezomib in patients with renal impairment. *Hematology* (2011) 16(4):200-8. Epub 2011/07/16. doi: 10.1179/102453311X13025568941880. PubMed PMID: 21756535.

31. Millennium Pharmaceuticals. Velcade [package insert]. U.S. Food and Drug Administration, <https://www.accessdata.fda.gov/drugsatfda_docs/label/2005/021602s006lbl.pdf>. Revised 3/2005. Accessed 6/2021. .

32. Wellbutrin [package insert]. Research Triangle Park, NC. GlaxoSmithKline; 2017.

33. Daviss WB, Perel JM, Rudolph GR, Axelson DA, Gilchrist R, Nuss S, et al. Steady-state pharmacokinetics of bupropion SR in juvenile patients. *J Am Acad Child Adolesc Psychiatry* (2005) 44(4):349-57. Epub 2005/03/23. doi: 10.1097/01.chi.0000153225.26850.26. PubMed PMID: 15782082.

34. Product Information: Aplenzin(TM) oral extended-release tablet, bupropion hydrobromide oral extended-release tablet. BTA Pharmaceuticals (per FDA), Bridgewater, NJ, 2011.

35. Product Information: TEGRETOL®, carbamazepine oral tablets. Novartis Pharmaceuticals corporation. East Hanover, NJ. 2009.

36. Battino D, Estienne M, Avanzini G. Clinical pharmacokinetics of antiepileptic drugs in paediatric patients. Part II. Phenytoin, carbamazepine, sulthiame, lamotrigine, vigabatrin, oxcarbazepine and felbamate. *Clin Pharmacokinet* (1995) 29(5):341-69. Epub 1995/11/01. doi: 10.2165/00003088-199529050-00004. PubMed PMID: 8582119.

37. Cancidas [package insert]. Whitehouse Station, NJ. Merck; 2005.

38. Product Information: MAXIPIME intravenous injection, intramuscular injection, cefepime HCl intravenous injection, intramuscular injection. Hospira, Inc. (per manufacturer), Lake Forest, IL, 2018.

39. Product Information: CELEBREX®, celecoxib oral tablets. Pfizer Inc. New York, NY. 2018.

40. Bikle DD. Vitamin D metabolism, mechanism of action, and clinical applications. *Chem Biol* (2014) 21(3):319-29. Epub 2014/02/18. doi: 10.1016/j.chembiol.2013.12.016. PubMed PMID: 24529992; PubMed Central PMCID: PMCPMC3968073.

41. Adakveo [package insert]. East Hanover, NJ. Novartis Pharmaceuticals.; 2019.

42. Broniscer A, Jia S, Mandrell B, Hamideh D, Huang J, Onar-Thomas A, et al. Phase 1 trial, pharmacokinetics, and pharmacodynamics of dasatinib combined with crizotinib in children with recurrent or progressive high-grade and diffuse intrinsic pontine glioma. *Pediatr Blood Cancer* (2018) 65(7):e27035. Epub 2018/03/08. doi: 10.1002/pbc.27035. PubMed PMID: 29512900; PubMed Central PMCID: PMCPMC5980705.

43. Xalkori [package insert]. New York, NY. Pfizer; 2016.

44. Hoppu K, Koskimies O, Holmberg C, Hirvisalo EL. Pharmacokinetically determined cyclosporine dosage in young children. *Pediatr Nephrol* (1991) 5(1):1-4. Epub 1991/01/01. doi: 10.1007/BF00852828. PubMed PMID: 2025515.

45. Product Information: Sandimmune(R) oral soft gelatin capsules, oral solution, intravenous injection, cyclosporine oral soft gelatin capsules, oral solution, intravenous injection. Novartis Pharmaceuticals Corporation, East Hanover, NJ, 2013.

46. Periclou AP, Avramis VI. NONMEM population pharmacokinetic studies of cytosine arabinoside after high-dose and after loading bolus followed by continuous infusion of the drug in pediatric patients with leukemias. *Cancer Chemother Pharmacol* (1996) 39(1-2):42-50. Epub 1996/01/01. doi: 10.1007/s002800050536. PubMed PMID: 8995498.

47. Product Information: Cytosar-U(R), cytarabine. Upjohn Company, Kalamazoo, MI, USA, 1999.

48. Mirochnick M, Michaels M, Clarke D, Brena A, Regan AM, Pelton S. Pharmacokinetics of dapsone in children. *J Pediatr* (1993) 122(5 Pt 1):806-9. Epub 1993/05/01. doi: 10.1016/s0022-3476(06)80033-8. PubMed PMID: 8496767.

49. Wozel G, Blasum C. Dapsone in dermatology and beyond. *Arch Dermatol Res* (2014) 306(2):103-24. Epub 2013/12/07. doi: 10.1007/s00403-013-1409-7. PubMed PMID: 24310318; PubMed Central PMCID: PMCPMC3927068.

50. Darzalex [package insert]. Horsham, PA. Janssen Pharmaceutical Company; 2015.

51. Christopher LJ, Cui D, Wu C, Luo R, Manning JA, Bonacorsi SJ, et al. Metabolism and disposition of dasatinib after oral administration to humans. *Drug Metab Dispos* (2008) 36(7):1357-64. Epub 2008/04/19. doi: 10.1124/dmd.107.018267. PubMed PMID: 18420784.

52. Li X, He Y, Ruiz CH, Koenig M, Cameron MD, Vojkovsky T. Characterization of dasatinib and its structural analogs as CYP3A4 mechanism-based inactivators and the proposed bioactivation pathways. *Drug Metab Dispos* (2009) 37(6):1242-50. Epub 2009/03/14. doi: 10.1124/dmd.108.025932. PubMed PMID: 19282395; PubMed Central PMCID: PMCPMC3202349.

53. Zwaan CM, Rizzari C, Mechinaud F, Lancaster DL, Lehrnbecher T, van der Velden VH, et al. Dasatinib in children and adolescents with relapsed or refractory leukemia: results of the CA180-018 phase I dose-escalation study of the Innovative Therapies for Children with Cancer Consortium. *J Clin Oncol* (2013) 31(19):2460-8. Epub 2013/05/30. doi: 10.1200/JCO.2012.46.8280. PubMed PMID: 23715577.

54. Tanaka C. Clinical pharmacology of deferasirox. *Clin Pharmacokinet* (2014) 53(8):679-94. Epub 2014/07/06. doi: 10.1007/s40262-014-0151-4. PubMed PMID: 24996374.

55. Kwiatkowski J, Duffner U, Abdel-Mageed A. Deferasirox Decreases Busulfan Clearance. *Ann Pharmacother* (2018) 52(5):497-8. Epub 2018/01/11. doi: 10.1177/1060028017752920. PubMed PMID: 29319328.

56. Sweiss K, Patel P, Rondelli D. Deferasirox increases BU blood concentrations. *Bone Marrow Transplant* (2012) 47(2):315-6. Epub 2011/04/05. doi: 10.1038/bmt.2011.75. PubMed PMID: 21460865.

57. Fassos FF, Klein J, Fernandes D, Matsui D, Olivieri NF, Koren G. The pharmacokinetics and pharmacodynamics of the oral iron chelator deferiprone (L1) in relation to hemoglobin levels. *Int J Clin Pharmacol Ther* (1996) 34(7):288-92. Epub 1996/07/01. PubMed PMID: 8832304.

58. Product Information: DESFERAL(R) Injection Route Powder for Solution, deferoxamine mesylate injection route powder for solution. Novartis Pharmaceuticals Corporation, East Hanover, NJ, 2002.

59. Pichard L, Fabre I, Daujat M, Domergue J, Joyeux H, Maurel P. Effect of corticosteroids on the expression of cytochromes P450 and on cyclosporin A oxidase activity in primary cultures of human hepatocytes. *Mol Pharmacol* (1992) 41(6):1047-55. Epub 1992/06/01. PubMed PMID: 1614409.

60. Richter O, Ern B, Reinhardt D, Becker B. Pharmacokinetics of dexamethasone in children. *Pediatr Pharmacol (New York)* (1983) 3(3-4):329-37. Epub 1983/01/01. PubMed PMID: 6677878.

61. Decadron [package insert]. Whitehouse Station, NJ. Merck; 2009.

62. Bjornsson TD, Huang AT, Roth P, Jacob DS, Christenson R. Effects of high-dose cancer chemotherapy on the absorption of digoxin in two different formulations. *Clin Pharmacol Ther* (1986) 39(1):25-8. Epub 1986/01/01. doi: 10.1038/clpt.1986.4. PubMed PMID: 3943266.

63. Product Information: LANOXIN®, digoxin oral tablets. Concordia Pharmaceuticals Inc. St. Michael, Barbados. 2015.

64. Product Information: BENADRYL®, diphenhydramine oral and injection use. BD Rx Inc. Franlkin Lakes, NJ. 2012.

65. Watanabe K, Yamaori S, Funahashi T, Kimura T, Yamamoto I. Cytochrome P450 enzymes involved in the metabolism of tetrahydrocannabinols and cannabinol by human hepatic microsomes. *Life Sci* (2007) 80(15):1415-9. Epub 2007/02/17. doi: 10.1016/j.lfs.2006.12.032. PubMed PMID: 17303175.

66. Marinol [package insert]. North Chicago, IL. AbbVie Inc.; 2017.

67. Lobo ED, Quinlan T, Prakash A. Pharmacokinetics of orally administered duloxetine in children and adolescents with major depressive disorder. *Clin Pharmacokinet* (2014) 53(8):731-40. Epub 2014/07/06. doi: 10.1007/s40262-014-0149-y. PubMed PMID: 24989060.

68. Product Information: CYMBALTA®, duloxetine capsules for oral use. Eli Lilly and Company. Indianapolis, IN. 2010.

69. Promacta [package insert]. Research Triangle Park, NC. GlaxoSmithKline; 2015.

70. Lambert MP. Spotlight on eltrombopag in the treatment of children with chronic immune thrombocytopenia. *Pediatric Health Med Ther* (2016) 7:39-43. Epub 2016/06/08. doi: 10.2147/PHMT.S90688. PubMed PMID: 29388634; PubMed Central PMCID: PMCPMC5683296.

71. Product Information: GAMIFANT®, emapalumab-izsg injection for IV use. Novimmune SA. Geneva, Switzerland. 2018.

72. Product Information: LOVENOX®, enoxaparin sodium injection for subcutaneous and IV use. Sanofi-aventis U.S. LLC. Bridgewater, NJ. 2009.

73. Sinkule JA, Hutson P, Hayes FA, Etcubanas E, Evans W. Pharmacokinetics of etoposide (VP16) in children and adolescents with refractory solid tumors. *Cancer Res* (1984) 44(7):3109-13. Epub 1984/07/01. PubMed PMID: 6539169.

74. Yang J, Bogni A, Schuetz EG, Ratain M, Dolan ME, McLeod H, et al. Etoposide pathway. *Pharmacogenet Genomics* (2009) 19(7):552-3. Epub 2009/06/11. doi: 10.1097/FPC.0b013e32832e0e7f. PubMed PMID: 19512958; PubMed Central PMCID: PMCPMC4164627.

75. Brammer KW, Coates PE. Pharmacokinetics of fluconazole in pediatric patients. *Eur J Clin Microbiol Infect Dis* (1994) 13(4):325-9. Epub 1994/04/01. doi: 10.1007/BF01974613. PubMed PMID: 8070441.

76. Glotzbecker B, Duncan C, Alyea E, 3rd, Campbell B, Soiffer R. Important drug interactions in hematopoietic stem cell transplantation: what every physician should know. *Biol Blood Marrow Transplant* (2012) 18(7):989-1006. Epub 2011/12/14. doi: 10.1016/j.bbmt.2011.11.029. PubMed PMID: 22155504.

77. Product Information: PROZAC®, fluoxetine capsules for oral use. Lilly USA, LLC. Indianapolis, IN. 2017.

78. Product Information: LUVOX®, fluvoxamine maleate tablets for oral use. ANI pharmaceuticals, Inc. Baudette, MN. 2012.

79. Product Information: EMEND®, fosaprepitant injection for IV use. Merck& Co., inc. Whitehouse Station, NJ. 2018.

80. Product Information: CEREBYX®, fosphenytoin sodium injection for IV or IM use. Pfizer Inc. New York, NY. 2017.

81. Product Information: NEURONTIN®, gabapentin tablets, capsules, and solution for oral use. Pfizer Inc. New York, NY. 2017.

82. Haig GM, Bockbrader HN, Wesche DL, Boellner SW, Ouellet D, Brown RR, et al. Single-dose gabapentin pharmacokinetics and safety in healthy infants and children. *J Clin Pharmacol* (2001) 41(5):507-14. Epub 2001/05/22. doi: 10.1177/00912700122010384. PubMed PMID: 11361047.

83. Cytovene [package insert]. South San Francisco, CA. Genentech; 2017.

84. Product Information: MYLOTARG®, gemtuzumab-ozogamicin injection for IV use. Pfizer Inc. New York, NY. 2017.

85. Buckwalter M, Dowell JA, Korth-Bradley J, Gorovits B, Mayer PR. Pharmacokinetics of gemtuzumab ozogamicin as a single-agent treatment of pediatric patients with refractory or relapsed acute myeloid leukemia. *J Clin Pharmacol* (2004) 44(8):873-80. Epub 2004/08/03. doi: 10.1177/0091270004267595. PubMed PMID: 15286091.

86. Cortes JE, de Lima M, Dombret H, Estey EH, Giralt SA, Montesinos P, et al. Prevention, recognition, and management of adverse events associated with gemtuzumab ozogamicin use in acute myeloid leukemia. *J Hematol Oncol* (2020) 13(1):137. Epub 2020/10/17. doi: 10.1186/s13045-020-00975-2. PubMed PMID: 33059764; PubMed Central PMCID: PMCPMC7559451.

87. Pautas C, Raffoux E, Lambert J, Legrand O, Chantepie S, Gastaud L, et al. Outcomes following hematopoietic stem cell transplantation in patients treated with standard chemotherapy with or without gemtuzumab ozogamicin for acute myeloid leukemia. *Bone Marrow Transplant* (2021). Epub 2021/02/11. doi: 10.1038/s41409-020-01207-4. PubMed PMID: 33564120.

88. Xospata [package insert]. Northbrook, IL.Astellas Pharma US, Inc; 2015.

89. Levis M, Smith C, Litzow M, Perl A, Altman JK, James A, et al. Drug-Drug Interaction Potential of Gilterinitib in Healthy Subjects and Patients with Relapsed /Refractory Acute Myeloid Leukemia. *Abstract* (2017).

90. Product Information: ENDARI(TM) oral powder, L-glutamine oral powder. Emmaus Medical, Inc, Torrance, CA, 2017.

91. Kytril [package insert]. Nutley, NJ. Roche Laboratories Inc.; 2009.

92. Melin J, Parra-Guillen ZP, Michelet R, Truong T, Huisinga W, Hartung N, et al. Pharmacokinetic/Pharmacodynamic Evaluation of Hydrocortisone Therapy in Pediatric Patients with Congenital Adrenal Hyperplasia. *J Clin Endocrinol Metab* (2020) 105(3). Epub 2020/02/14. doi: 10.1210/clinem/dgaa071. PubMed PMID: 32052005.

93. Estepp JH, Wiczling P, Moen J, Kang G, Mack JM, Liem R, et al. Hydroxycarbamide in children with sickle cell anaemia after first-dose vs. chronic therapy: pharmacokinetics and predictive models for drug exposure. *Br J Clin Pharmacol* (2018) 84(7):1478-85. Epub 2017/09/09. doi: 10.1111/bcp.13426. PubMed PMID: 28884840; PubMed Central PMCID: PMCPMC6005595.

94. Ware RE, Despotovic JM, Mortier NA, Flanagan JM, He J, Smeltzer MP, et al. Pharmacokinetics, pharmacodynamics, and pharmacogenetics of hydroxyurea treatment for children with sickle cell anemia. *Blood* (2011) 118(18):4985-91. Epub 2011/08/31. doi: 10.1182/blood-2011-07-364190. PubMed PMID: 21876119; PubMed Central PMCID: PMCPMC3208303.

95. Droxia [package insert]. Princeton, NJ. Bristol-Myers Squibb Company; 2010.

96. Gleevec [package insert]. East Hanover, NJ. Novartis Pharmaceuticals Corporation; 2008.

97. Product Information: BESPONSA®, inotuzumab-ozogamicin injection for IV use. Pfizer Inc. Philadelphia, PA. 2017.

98. Kantarjian HM, DeAngelo DJ, Stelljes M, Martinelli G, Liedtke M, Stock W, et al. Inotuzumab Ozogamicin versus Standard Therapy for Acute Lymphoblastic Leukemia. *N Engl J Med* (2016) 375(8):740-53. Epub 2016/06/14. doi: 10.1056/NEJMoa1509277. PubMed PMID: 27292104; PubMed Central PMCID: PMCPMC5594743.

99. Kebriaei P, Cutler C, de Lima M, Giralt S, Lee SJ, Marks D, et al. Management of important adverse events associated with inotuzumab ozogamicin: expert panel review. *Bone Marrow Transplant* (2018) 53(4):449-56. Epub 2018/01/14. doi: 10.1038/s41409-017-0019-y. PubMed PMID: 29330398; PubMed Central PMCID: PMCPMC5897380.

100. Yurkiewicz IR, Muffly L, Liedtke M. Inotuzumab ozogamicin: a CD22 mAb-drug conjugate for adult relapsed or refractory B-cell precursor acute lymphoblastic leukemia. *Drug Des Devel Ther* (2018) 12:2293-300. Epub 2018/08/09. doi: 10.2147/DDDT.S150317. PubMed PMID: 30087554; PubMed Central PMCID: PMCPMC6063246.

101. Groll AH, Desai A, Han D, Howieson C, Kato K, Akhtar S, et al. Pharmacokinetic Assessment of Drug-Drug Interactions of Isavuconazole With the Immunosuppressants Cyclosporine, Mycophenolic Acid, Prednisolone, Sirolimus, and Tacrolimus in Healthy Adults. *Clin Pharmacol Drug Dev* (2017) 6(1):76-85. Epub 2016/06/09. doi: 10.1002/cpdd.284. PubMed PMID: 27273343; PubMed Central PMCID: PMCPMC5298005.

102. Cresemba [package insert]. Northbrook, IL. Astellas Pharma US, Inc; 2015.

103. Product Information: ACCUTANE®, isotretinoin capsules for oral use. Roche Laboratories Inc. Nutley, NJ. 2002.

104. Sporanox [package insert]. Research Triangle Park, NC. Stiefel; 2010.

105. de Repentigny L, Ratelle J, Leclerc JM, Cornu G, Sokal EM, Jacqmin P, et al. Repeated-dose pharmacokinetics of an oral solution of itraconazole in infants and children. *Antimicrob Agents Chemother* (1998) 42(2):404-8. Epub 1998/04/04. PubMed PMID: 9527794; PubMed Central PMCID: PMCPMC105422.

106. Product Information: KETALAR®, ketamine hydrochloride injection. JHP Pharmaceuticals, LLC. Rochester, MI. 2012.

107. Product Information: ALDURAZYME®, laronidase injection for IV use. BioMarin Pharmaceutical Inc. Novato, CA. 2010.

108. Chien S, Wells TG, Blumer JL, Kearns GL, Bradley JS, Bocchini JA, Jr., et al. Levofloxacin pharmacokinetics in children. *J Clin Pharmacol* (2005) 45(2):153-60. Epub 2005/01/14. doi: 10.1177/0091270004271944. PubMed PMID: 15647407.

109. Jones CB, Fugate SE. Levofloxacin and warfarin interaction. *Ann Pharmacother* (2002) 36(10):1554-7. Epub 2002/09/24. doi: 10.1345/aph.1C074. PubMed PMID: 12243605.

110. Product Information: Levofloxacin oral solution, levofloxacin oral solution. Lannett Company Inc. (per DailyMed), Philadelphia, PA, 2018.

111. Product Information: ATIVAN®, lorazepam tablets for oral use. Valeant Pharmaceuticals North America LLC. Bridgewater, NJ. 2016.

112. McDermott CA, Kowalczyk AL, Schnitzler ER, Mangurten HH, Rodvold KA, Metrick S. Pharmacokinetics of lorazepam in critically ill neonates with seizures. *J Pediatr* (1992) 120(3):479-83. Epub 1992/03/01. doi: 10.1016/s0022-3476(05)80925-4. PubMed PMID: 1538303.

113. Product Information: Megace(R), megestrol acetate tablets, USP. Bristol-Myers Oncology Division, Princeton, NJ, 2001.

114. House L, Seminerio MJ, Mirkov S, Ramirez J, Skor M, Sachleben JR, et al. Metabolism of megestrol acetate in vitro and the role of oxidative metabolites. *Xenobiotica* (2018) 48(10):973-83. Epub 2017/10/21. doi: 10.1080/00498254.2017.1386335. PubMed PMID: 29050522; PubMed Central PMCID: PMCPMC6129397.

115. Christensson BA, Nilsson-Ehle I, Hutchison M, Haworth SJ, Oqvist B, Norrby SR. Pharmacokinetics of meropenem in subjects with various degrees of renal impairment. *Antimicrob Agents Chemother* (1992) 36(7):1532-7. Epub 1992/07/01. doi: 10.1128/aac.36.7.1532. PubMed PMID: 1510451; PubMed Central PMCID: PMCPMC191616.

116. Smith PB, Cohen-Wolkowiez M, Castro LM, Poindexter B, Bidegain M, Weitkamp JH, et al. Population pharmacokinetics of meropenem in plasma and cerebrospinal fluid of infants with suspected or complicated intra-abdominal infections. *Pediatr Infect Dis J* (2011) 30(10):844-9. Epub 2011/08/11. doi: 10.1097/INF.0b013e31822e8b0b. PubMed PMID: 21829139; PubMed Central PMCID: PMCPMC3173561.

117. Product Information: MERREM® IV, meropenem for injection. AztraZeneca Pharmaceuticals LP, Wilmington, DE. 2016.

118. Product Information: DOLOPHINE®, methadone hydrochloride tablets for oral use. West-Ward Pharmaceuticals Corp. Eatontown, NJ. 2018.

119. Bratlid D, Moe PJ. Pharmacokinetics of high-dose methotrexate treatment in children. *Eur J Clin Pharmacol* (1978) 14(2):143-7. Epub 1978/11/16. doi: 10.1007/BF00607446. PubMed PMID: 720376.

120. Forster VJ, van Delft FW, Baird SF, Mair S, Skinner R, Halsey C. Drug interactions may be important risk factors for methotrexate neurotoxicity, particularly in pediatric leukemia patients. *Cancer Chemother Pharmacol* (2016) 78(5):1093-6. Epub 2016/10/28. doi: 10.1007/s00280-016-3153-0. PubMed PMID: 27659182; PubMed Central PMCID: PMCPMC5083755.

121. Product Information: REDITREX®, methotrexate. Cumberland Pharmaceuticals Inc., Nashville,TN. 2019.

122. Product Information: REGLAN®, metoclopramide tablets for oral use and injection. Ani Pharmaceuticals, Inc. Baudette, MN. 2017.

123. Cohen-Wolkowiez M, Sampson M, Bloom BT, Arrieta A, Wynn JL, Martz K, et al. Determining population and developmental pharmacokinetics of metronidazole using plasma and dried blood spot samples from premature infants. *Pediatr Infect Dis J* (2013) 32(9):956-61. Epub 2013/04/17. doi: 10.1097/INF.0b013e3182947cf8. PubMed PMID: 23587979; PubMed Central PMCID: PMCPMC3769518.

124. Jager-Roman E, Doyle PE, Baird-Lambert J, Cvejic M, Buchanan N. Pharmacokinetics and tissue distribution of metronidazole in the new born infant. *J Pediatr* (1982) 100(4):651-4. Epub 1982/04/01. doi: 10.1016/s0022-3476(82)80779-8. PubMed PMID: 7062220.

125. Lamp KC, Freeman CD, Klutman NE, Lacy MK. Pharmacokinetics and pharmacodynamics of the nitroimidazole antimicrobials. *Clin Pharmacokinet* (1999) 36(5):353-73. Epub 1999/06/29. doi: 10.2165/00003088-199936050-00004. PubMed PMID: 10384859.

126. Templeton, editor. Metabolism and pharmacokinetics of metronidazole: a review. *Proceedings of the International Metronidazole Conference*; 1977; Montreal: Excerpta Medica.

127. He H, Tran P, Gu H, Tedesco V, Zhang J, Lin W, et al. Midostaurin, a Novel Protein Kinase Inhibitor for the Treatment of Acute Myelogenous Leukemia: Insights from Human Absorption, Metabolism, and Excretion Studies of a BDDCS II Drug. *Drug Metab Dispos* (2017) 45(5):540-55. Epub 2017/03/09. doi: 10.1124/dmd.116.072744. PubMed PMID: 28270565.

128. Yin OQ, Wang Y, Schran H. A mechanism-based population pharmacokinetic model for characterizing time-dependent pharmacokinetics of midostaurin and its metabolites in human subjects. *Clin Pharmacokinet* (2008) 47(12):807-16. Epub 2008/11/26. doi: 10.2165/0003088-200847120-00005. PubMed PMID: 19026036.

129. Rydapt [Package Insert]. East Hanover, NJ. Novartis Pharmaceuticals Corporation. 2017.

130. Product Information: MYCAMINE®, micafungin sodium for injection, IV use only. Astellas Pharma US, Inc. Deerfield, IL. 2011.

131. Heresi GP, Gerstmann DR, Reed MD, van den Anker JN, Blumer JL, Kovanda L, et al. The pharmacokinetics and safety of micafungin, a novel echinocandin, in premature infants. *Pediatr Infect Dis J* (2006) 25(12):1110-5. Epub 2006/11/30. doi: 10.1097/01.inf.0000245103.07614.e1. PubMed PMID: 17133155.

132. Walsh TJ, Goutelle S, Jelliffe RW, Golden JA, Little EA, DeVoe C, et al. Intrapulmonary pharmacokinetics and pharmacodynamics of micafungin in adult lung transplant patients. *Antimicrob Agents Chemother* (2010) 54(8):3451-9. Epub 2010/05/05. doi: 10.1128/AAC.01647-09. PubMed PMID: 20439610; PubMed Central PMCID: PMCPMC2916355.

133. Product Information: PRIMACOR®, milrinone lactate injection for IV use. Sanofi-Synthelabo Inc. North Chicago, IL. 2018.

134. Product Information: TASIGNA®, nilotinib capsules for oral use. Novartis Pharmaceuticals Corporation. East Hanover, NJ. 2010.

135. Product Information: ALINIA®, nitazoxanide tablets for oral use. Romark, L.C. Tampa, FL. 2016.

136. Product Information: ZYPREXA®, olanzapine tablet for oral use. Lilly USA, LLC. Indianapolis, IN. 2009. .

137. Grothe DR, Calis KA, Jacobsen L, Kumra S, DeVane CL, Rapoport JL, et al. Olanzapine pharmacokinetics in pediatric and adolescent inpatients with childhood-onset schizophrenia. *J Clin Psychopharmacol* (2000) 20(2):220-5. Epub 2000/04/19. doi: 10.1097/00004714-200004000-00015. PubMed PMID: 10770461.

138. Product Information: ZOFRAN®, ondansetron oral tablets and IV injection. GlaxoSmithKline, Research Triangle Park, NC, 2016.

139. Product Information: TRILEPTAL®, oxcarbazepine tablets for oral use. Novartis Pharmaceuticals Corporation. East Hanover, NJ. 2017.

140. Product Information: PROTONIX®, pantoprazole tablets for oral use. Pfizer Inc. Philadelphia, PA. 2012.

141. Paxil [package insert]. Research Triangle Park, NC. GlaxoSmithKline; 2012.

142. Findling RL, Reed MD, Myers C, O'Riordan MA, Fiala S, Branicky L, et al. Paroxetine pharmacokinetics in depressed children and adolescents. *J Am Acad Child Adolesc Psychiatry* (1999) 38(8):952-9. Epub 1999/08/06. doi: 10.1097/00004583-199908000-00010. PubMed PMID: 10434486.

143. Burenheide A, Kunze T, Clement B. Inhibitory effects on cytochrome p450 enzymes of pentamidine and its amidoxime pro-drug. *Basic Clin Pharmacol Toxicol* (2008) 103(1):61-5. Epub 2008/03/19. doi: 10.1111/j.1742-7843.2008.00236.x. PubMed PMID: 18346045.

144. Product Information: NebuPent(R) oral inhalation, pentamidine isethionate oral inhalation. APP Pharmaceuticals, LLC, Schaumburg, IL, 2011.

145. Product Information: DILANTIN®, phenytoin capsules for oral use. Pfizer Inc. New York, NY. 2009.

146. Pacifici. Clinical pharmacology of piperacillin-tazobactam combination in infants and children. *Clinical and Medical Investigations* (2019) 4:1-13.

147. Price KE, Saleem N, Lee G, Steinberg M. Potential of ponatinib to treat chronic myeloid leukemia and acute lymphoblastic leukemia. *Onco Targets Ther* (2013) 6:1111-8. Epub 2013/08/30. doi: 10.2147/OTT.S36980. PubMed PMID: 23986642; PubMed Central PMCID: PMCPMC3754816.

148. Iclusig [package insert]. Cambridge, MA. Ariad; 2012.

149. Product Information: Noxafil(R) oral delayed-release tablets, oral suspension, posaconazole oral delayed-release tablets, oral suspension. Merck Sharp & Dohme Corp. (per FDA), Whitehouse Station, NJ, 2013.

150. Bernardo VA, Cross SJ, Crews KR, Flynn PM, Hoffman JM, Knapp KM, et al. Posaconazole therapeutic drug monitoring in pediatric patients and young adults with cancer. *Ann Pharmacother* (2013) 47(7-8):976-83. Epub 2013/06/06. doi: 10.1345/aph.1R775. PubMed PMID: 23737511; PubMed Central PMCID: PMCPMC4384822.

151. Gustavson LE, Schweitzer SM, Koehne-Voss S, Achari R, Chira TO, Esslinger HU, et al. The effects of multiple doses of fenofibrate on the pharmacokinetics of pravastatin and its 3alpha-hydroxy isomeric metabolite. *J Clin Pharmacol* (2005) 45(8):947-53. Epub 2005/07/20. doi: 10.1177/0091270005278085. PubMed PMID: 16027406.

152. Hedman M, Neuvonen PJ, Neuvonen M, Antikainen M. Pharmacokinetics and pharmacodynamics of pravastatin in children with familial hypercholesterolemia. *Clin Pharmacol Ther* (2003) 74(2):178-85. Epub 2003/08/02. doi: 10.1016/S0009-9236(03)00153-X. PubMed PMID: 12891228.

153. Orapred ODT [package insert]. Atlanta, GA. Shionogi Pharma; 1955.

154. Rayos [package insert]. Deerfield, IL. Horizon Pharma; 1955.

155. Ferry JJ, Horvath AM, Bekersky I, Heath EC, Ryan CF, Colburn WA. Relative and absolute bioavailability of prednisone and prednisolone after separate oral and intravenous doses. *J Clin Pharmacol* (1988) 28(1):81-7. Epub 1988/01/01. doi: 10.1002/j.1552-4604.1988.tb03105.x. PubMed PMID: 3350994.

156. Product Information: LYRICA®, pregabalin capsules for oral use. Pfizer Inc. New York, NY. 2018.

157. Pfizer. Mycobutin [package insert], ]. U.S. Food and Drug Administration website <https://www.accessdata.fda.gov/drugsatfda_docs/label/2008/050689s016lbl.pdf> Revised Oct 2014, Accessed May 2021.

158. Product Information: RIFADIN(R) IV intravenous injection, rifampin intravenous injection. Sanofi-aventis US LLC (per FDA), Bridgewater, NJ, 2019.

159. Product Information: TRANSDERM SCOP®, scopolamine transdermal patch. Novartis Consumer Health, Inc. Parsippany, NJ. 2013.

160. Alderman J, Wolkow R, Chung M, Johnston HF. Sertraline treatment of children and adolescents with obsessive-compulsive disorder or depression: pharmacokinetics, tolerability, and efficacy. *J Am Acad Child Adolesc Psychiatry* (1998) 37(4):386-94. Epub 1998/04/29. doi: 10.1097/00004583-199804000-00016. PubMed PMID: 9549959.

161. DeVane CL, Liston HL, Markowitz JS. Clinical pharmacokinetics of sertraline. *Clin Pharmacokinet* (2002) 41(15):1247-66. Epub 2002/11/28. doi: 10.2165/00003088-200241150-00002. PubMed PMID: 12452737.

162. Hemeryck A, Belpaire FM. Selective serotonin reuptake inhibitors and cytochrome P-450 mediated drug-drug interactions: an update. *Curr Drug Metab* (2002) 3(1):13-37. Epub 2002/03/06. doi: 10.2174/1389200023338017. PubMed PMID: 11876575.

163. Goyal RK, Han K, Wall DA, Pulsipher MA, Bunin N, Grupp SA, et al. Sirolimus pharmacokinetics in early postmyeloablative pediatric blood and marrow transplantation. *Biol Blood Marrow Transplant* (2013) 19(4):569-75. Epub 2012/12/26. doi: 10.1016/j.bbmt.2012.12.015. PubMed PMID: 23266742; PubMed Central PMCID: PMCPMC4231793.

164. Product Information: RAPAMUNE oral solution oral tablets, sirolimus oral solution oral tablets. Pfizer, Inc. (per manufacturer), Philadelphia, PA, 2015.

165. Widemann BC, Kim A, Fox E, Baruchel S, Adamson PC, Ingle AM, et al. A phase I trial and pharmacokinetic study of sorafenib in children with refractory solid tumors or leukemias: a Children's Oncology Group Phase I Consortium report. *Clin Cancer Res* (2012) 18(21):6011-22. Epub 2012/09/11. doi: 10.1158/1078-0432.CCR-11-3284. PubMed PMID: 22962440; PubMed Central PMCID: PMCPMC4008314.

166. Nexavar [package insert]. Whippany, NJ. Bayer HealthCare Pharmaceuticals; 2005. .

167. Hoppu K, Koskimies O, Tuomisto J. Trimethoprim pharmacokinetics in children with renal insufficiency. *Clin Pharmacol Ther* (1987) 42(2):181-6. Epub 1987/08/01. doi: 10.1038/clpt.1987.130. PubMed PMID: 3301152.

168. Prograf [package insert]. Deerfield, IL. Astellas Pharma; 2012.

169. Wallemacq PE, Verbeeck RK. Comparative clinical pharmacokinetics of tacrolimus in paediatric and adult patients. *Clin Pharmacokinet* (2001) 40(4):283-95. Epub 2001/05/23. doi: 10.2165/00003088-200140040-00004. PubMed PMID: 11368293.

170. Lugowska I, Kosela-Paterczyk H, Kozak K, Rutkowski P. Trametinib: a MEK inhibitor for management of metastatic melanoma. *Onco Targets Ther* (2015) 8:2251-9. Epub 2015/09/09. doi: 10.2147/OTT.S72951. PubMed PMID: 26347206; PubMed Central PMCID: PMCPMC4556032.

171. Salama AK, Kim KB. MEK inhibition in the treatment of advanced melanoma. *Curr Oncol Rep* (2013) 15(5):473-82. Epub 2013/08/27. doi: 10.1007/s11912-013-0336-2. PubMed PMID: 23975010.

172. Mekinist [package insert]. East Hanover, NJ. Novartis Pharmaceuticals Corporation; 2018.

173. Product Information: URSO FORTE®, ursodiol oral tablets. Axcan Scandipharm Inc. Birmingham, AL. 2007.

174. Nadal D, Leverger G, Sokal EM, Floret D, Perel Y, Leibundgut K, et al. An investigation of the steady-state pharmacokinetics of oral valacyclovir in immunocompromised children. *J Infect Dis* (2002) 186 Suppl 1:S123-30. Epub 2002/09/28. doi: 10.1086/342968. PubMed PMID: 12353197.

175. Product Information: VALTREX(R) oral caplets, valacyclovir hydrochloride oral caplets. GlaxoSmithKline, Research Triangle Park, NC, 2010.

176. Product Information: VALCYTE(R) oral solution, oral tablets, valganciclovir oral solution, oral tablets. Roche Laboratories Inc., Nutley, NJ, 2009.

177. Venclexta [package insert]. North Chicago, IL.AbbVie Inc.; 2020.

178. Groninger E, Meeuwsen-de Boar T, Koopmans P, Uges D, Sluiter W, Veerman A, et al. Pharmacokinetics of vincristine monotherapy in childhood acute lymphoblastic leukemia. *Pediatr Res* (2002) 52(1):113-8. Epub 2002/06/27. doi: 10.1203/00006450-200207000-00021. PubMed PMID: 12084857.

179. Product Information: vincristine sulfate IV Injection, vincristine sulfate IV Injection. Hospira, Inc., Lake Forest, IL, 2007.

180. Walsh TJ, Karlsson MO, Driscoll T, Arguedas AG, Adamson P, Saez-Llorens X, et al. Pharmacokinetics and safety of intravenous voriconazole in children after single- or multiple-dose administration. *Antimicrob Agents Chemother* (2004) 48(6):2166-72. Epub 2004/05/25. doi: 10.1128/AAC.48.6.2166-2172.2004. PubMed PMID: 15155217; PubMed Central PMCID: PMCPMC415618.

181. Oxbryta [package insert]. South San Francisco, CA. Global Blood Therapeutics, Inc. 2019.

182. Forest Laboratories. Lexapro [product insert]. U.S. Federal Drug Administration website. <https://www.accessdata.fda.gov/drugsatfda_docs/label/2009/021323s032,021365s023lbl.pdf>. Revised 1/2017. Accessed 5/2021.

183. Yilmaz Z, Ceschi A, Rauber-Luthy C, Sauer O, Stedtler U, Prasa D, et al. Escitalopram causes fewer seizures in human overdose than citalopram. *Clin Toxicol (Phila)* (2010) 48(3):207-12. Epub 2010/02/23. doi: 10.3109/15563650903585937. PubMed PMID: 20170390.

184. Center for Drug Evaluation and Research. (n.d.). Revised recommendations for Celexa. Retrieved from <https://www.fda.gov/drugs/drug-safety-and-availability/fda-drug-safety-communication-revised-recommendations-celexa-citalopram-hydrobromide-related>.

185. Viibryd [package insert]. New Haven, CT. Merck; 2010.

186. Trintellix [package insert]. Deerfield, IL. Takeda Pharmaceuticals America; 2013.

187. Remeron [package insert]. Kenilworth, NJ. Schering Corporation; 2007.

188. Effexor [package insert]. Philadelphia, PA. Wyeth Pharmaceuticals; 2017.
